# Electrostatics and Charge Regulation Drive Phosphate Accumulation in Cationic Dendrimers

**DOI:** 10.1021/acsapm.6c02186

**Published:** 2026-07-31

**Authors:** Pablo M. Blanco, Ganesh Balaji, Corinna Dannert, Sebastian P. Pineda, Carlos Rey-Castro, Josep Lluís Garcés, Peter Košovan, Rita S. Dias

**Affiliations:** † Department of Chemistry, Physics, Environmental and Soil Sciences, 16739 University of Lleida − AGROTECNIO-CERCA Center, Rovira Roure 191, Lleida 25198, Spain; ‡ Department of Physics, 8018 NTNU - Norwegian University of Science and Technology, Trondheim NO-7491, Norway; § Department of Chemical Engineering, Indian Institute of Technology Madras, IIT P.O., Sardar Patel Road, Adyar Chennai, Tamil Nadu 600036, India; ∥ Department of Physical and Macromolecular Chemistry, Faculty of Science, 37740 Charles University, Hlavova 2030, Prague 2, Prague 128 40, Czech Republic; ⊥ Department of Chemistry, Division of Computational Chemistry, Lund University, Naturvetarvägen 22, Lund 22100, Sweden

**Keywords:** PAMAM dendrimers, Charge regulation, Constant-pH molecular simulations, Phosphate buffer, Potentiometric titration, Branched polycations, Electrostatic interactions

## Abstract

Branched polycations such as poly­(amidoamine) (PAMAM) dendrimers have been extensively investigated for applications including drug delivery and gene transfection, where phosphate-buffered solutions are commonly employed to maintain pH. Experimental and simulation studies have reported phosphate accumulation around PAMAM and attributed it, at least in part, to phosphate-specific interactions. Here, we investigate whether such specific interactions are necessary to explain phosphate association. Combining constant-pH molecular simulations, potentiometric titrations, and Site Binding theory, we study the coupled ionization of G2 PAMAM dendrimers and phosphate ions in aqueous solution. By employing a coarse-grained model that intentionally excludes phosphate-specific short-range interactions, we isolate the effects of electrostatics and charge regulation. The simulations quantitatively reproduce the ionization behavior of PAMAM and phosphate and reveal a strongly asymmetric charge-regulation response: phosphate has only a minor effect on PAMAM ionization, whereas PAMAM significantly enhances phosphate ionization in its vicinity. This coupling drives phosphate accumulation around PAMAM, with maximum adsorption at circumneutral pH where both species are highly charged. At phosphate concentrations comparable to those used in phosphate-buffered saline, the adsorbed phosphates reduce the effective charge of the PAMAM−phosphate complex. The qualitative agreement with previous experimental observations demonstrates that phosphate accumulation does not necessarily require phosphate-specific interactions but can emerge as a generic consequence of electrostatic attraction and coupled ionization equilibria. More broadly, the results highlight that multivalent buffer ions can act as active regulators of macromolecular electrostatics rather than merely as pH-control agents or passive screening species.

## Graphical abstract

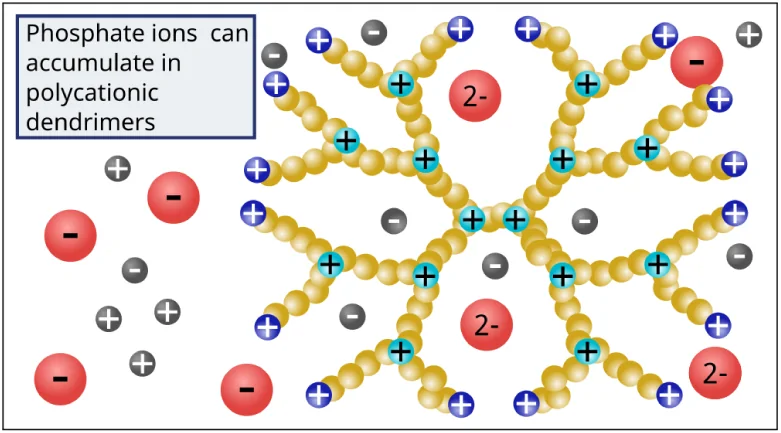

## 1 Introduction

Buffer ions are ubiquitous in biological and physicochemical experiments involving ionizable macromolecules. Among the available buffering systems, phosphate-buffered saline (PBS), composed of the $H_{2}{\mathrm{PO}}_{4}^{-}/{\mathrm{HPO}}_{4}^{2-}$ conjugate pair (p*K*_A_ = 7.21), is particularly widespread because of its physiological relevance, chemical stability, low cost, and reproducibility.^1–4^ Yet phosphate ions are often regarded as passive components whose primary role is to maintain pH, despite increasing evidence that they can directly influence the behavior of charged macromolecules.^5^

Applications governed by electrostatic interactions are inherently sensitive to the presence of phosphate ions, which are predominantly divalent under physiological conditions. Systems such as enzyme immobilization on charged surfaces^6–8^ and nucleic acid complexation with cationic lipids or polymers^9,10^ rely strongly on electrostatic attraction and counterion-release entropy. In these contexts, multivalent phosphate ions not only screen electrostatic interactions but can also compete for binding sites, reduce the counterion entropy gain upon complexation, and destabilize assembled structures by displacing weakly bound macromolecules or triggering premature disassembly.^11,12^

Beyond these generic electrostatic effects, a growing body of evidence indicates that buffer ions can directly influence the ionization, stability, mobility, self-assembly, and adsorption behavior of biomacromolecules.^5,13,14^ In proteins and related systems, such buffer effects are commonly interpreted as resulting from a delicate interplay among electrostatic, dispersion, hydration, and hydrogen-bonding interactions. For example, Parsons et al. showed that different buffer ions, including phosphate, can produce markedly different zeta potentials for lysozyme at the same pH and buffer concentration, and attributed these effects to ion-specific dispersion forces using a modified Poisson−Boltzmann model incorporating charge regulation at the protein surface.^15^ Notably, in that model the buffer acid−base composition was fixed by the bulk solution conditions, rather than explicitly coupled to the local electrostatic environment through charge regulation of the buffer ions themselves. This leaves open the question of how much of the observed buffer-specific behavior may originate from the coupled ionization of the buffer ions and the macromolecule. Overall, phosphate ions are increasingly recognized as active participants in macromolecular processes rather than merely pH-regulating species.

Within this broader context, branched polymeric vehicles have been extensively investigated for applications such as nucleic acid delivery^16–18^ drug delivery,^19^ and as antimicrobial therapies.^20^ Their high density of ionizable amine groups also confers a substantial buffering capacity, enabling proton uptake over a broad pH range, which is believed to facilitate endosomal escape in intracellular delivery.^21–25^ Among these systems, poly­(amidoamine) (PAMAM) dendrimers are attractive due to their well-defined monodisperse structure^26^ and tunable surface chemistry, enabling applications ranging from drug and nucleic acid delivery to water treatment.^26–29^

Several studies have reported phosphate accumulation around PAMAM dendrimers and concomitant changes in their physicochemical properties. Experimental measurements^30,31^ and atomistic simulations^32^ have shown that phosphate ions preferentially associate with PAMAM, and this behavior has been attributed, at least in part, to phosphate-specific interactions such as hydrogen bonding with tertiary amines.

Both PAMAM dendrimers and phosphate ions are weak electrolytes whose protonation states depend on pH and on the local electrostatic environment. As a result, their charges are not fixed but adapt dynamically through charge regulation, that is, the coupling between protonation equilibria and molecular interactions.^33–35^ Charge regulation is now recognized as a fundamental mechanism governing interactions in systems ranging from proteins and weak polyelectrolytes to soft interfaces and self-assembling materials.^36–47^ This raises the question of whether phosphate accumulation and the resulting charge renormalization of PAMAM can arise as a generic consequence of electrostatics and coupled ionization equilibria, even in the absence of phosphate-specific interactions. This question is particularly pertinent because phosphate is itself a titratable multivalent ion whose charge adapts to its local electrostatic environment. Such mutual charge regulation can modify ion distributions around the dendrimer and substantially alter the effective charge of the resulting PAMAM−phosphate complex.

In this work, we combine constant-pH simulations,^48^ Site Binding theory^49^ and potentiometric titrations to investigate the coupled ionization of phosphate ions and PAMAM dendrimers in aqueous solution. In contrast to previous atomistic simulation studies,^32^ our coarse-grained model intentionally excludes phosphate-specific short-range interactions, including hydrogen bonding, hydration, and dispersion forces, which allows us to isolate the contribution of electrostatics and coupled acid−base equilibria to phosphate accumulation. By doing so, we directly test whether the experimentally observed influence of phosphate on PAMAM requires phosphate-specific interactions, or can emerge from generic electrostatic mechanisms alone.

**Figure 1:**
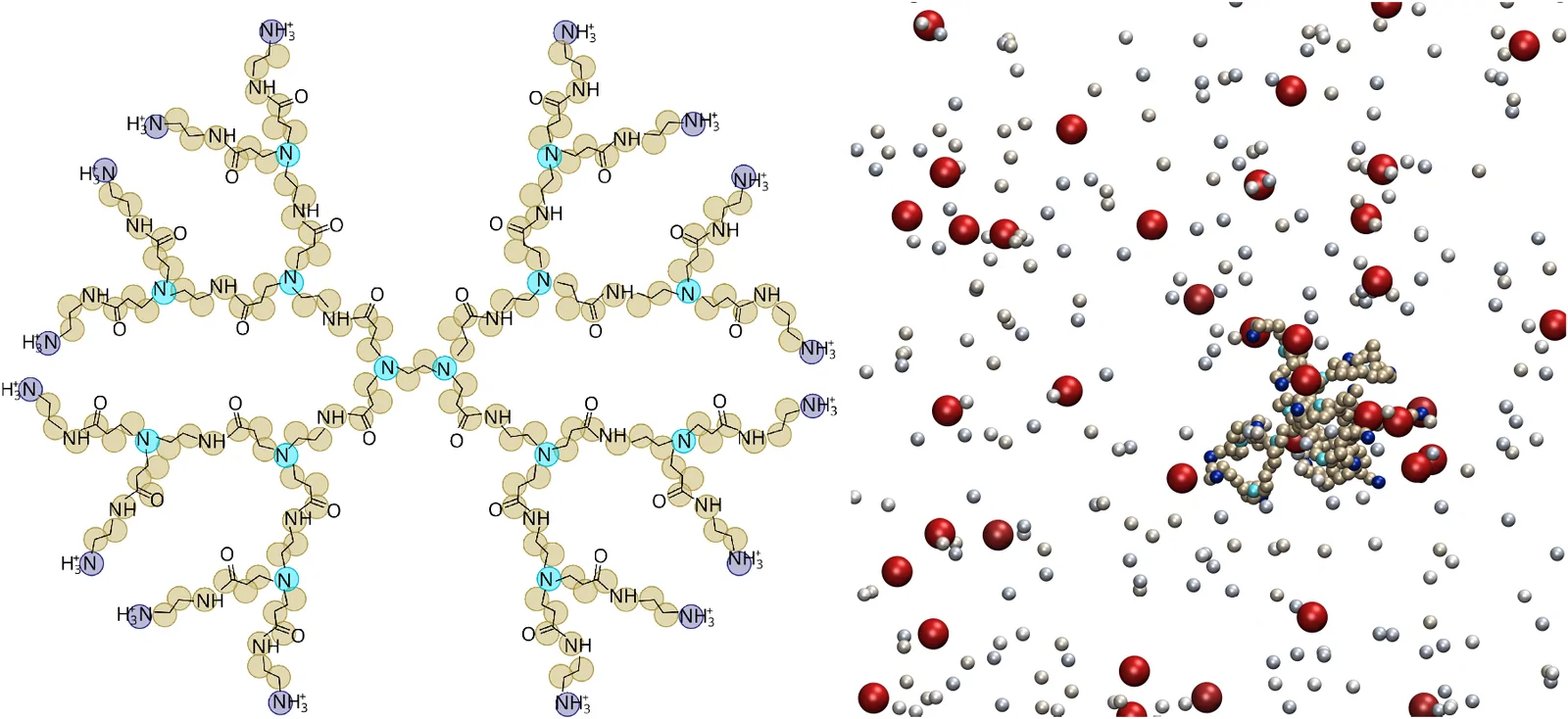
Left panel: Scheme of a generation 2 (G2) PAMAM. Right panel: Representative snapshot of the G2 PAMAM coarse-grained model in a phosphate buffer solution 49.4 mM at pH 7 with 100 mM salt concentration. Color code: PAMAM primary amines (purple), PAMAM tertiary amines (cyan), PAMAM inert groups (tin), phosphates (red), and monovalent salt ions (gray).

## 2 Theoretical Background in Ionization Equilibrium

To establish a reference for assessing interaction-induced effects on ionization, we employ the Henderson−Hasselbalch (HH) description of acid−base equilibria. In this idealized framework, each ionizable group titrates independently in infinitely dilute solution, such that molecular interactions are neglected and activities are approximated by concentrations. The ionization state of each group therefore depends only on the solution pH and its intrinsic (bare) p*K*_A_ value.

### 2.1 Monoprotic Bases: Amine Groups in PAMAM

As shown in Figure 1, generation 2 (G2) PAMAM contains *N*_amine_ = 30 amine groups: 16 primary ( $pK_{A}^{I}=9.0$ , purple spheres) and 14 tertiary ( $pK_{A}^{\mathrm{III}}=6.0$ , cyan spheres).^50^ Both types of amines are monoprotic bases that ionize according to the equilibria:

1 $$-{\mathrm{NH}}_{3}^{+}⇌-{\mathrm{NH}}_{2}+H^{+},\;\left(\mathrm{primary}\right)$$

2 $$-{\mathrm{NRH}}^{+}-⇌-\mathrm{NR}-+H^{+}\;\left(\mathrm{tertiary}\right)$$

where *R* denotes a generic aliphatic substituent. For a monoprotic group *i*, the fraction of the group in its charged state α_*i*_, also known as its ionization degree, is described by the Henderson−Hasselbalch (HH) equation,

3 $$\alpha_{i}=\frac{1}{1+10^{z\left(pK_{A}^{i}-\mathrm{pH}\right)}}$$

where pH = −log_10_ *a*_H^+^_, with *a*_H^+^_ denoting the proton activity, and $pK_{A}^{i}=-\log_{10}K_{A}^{i}$ , where $K_{A}^{i}$ is the thermodynamic acidity constant of group *i*. The parameter *z* defines the acid−base character of the ionizable group, with *z* = +1 for acidic groups and *z* = −1 for basic groups. The overall charge of PAMAM is then calculated according to

4 $$Q_{\mathrm{PAMAM}}=e\overset{N_{\mathrm{amine}}}{\underset{i=1}{\sum }}\alpha_{i}$$

where the summation extends over all amine groups and *e* is the elementary charge.

### 2.2 Polyprotic Acids: Phosphate

In contrast to monoprotic species, phosphate undergoes three successive deprotonation reactions,

5 $$H_{3}{\mathrm{PO}}_{4}⇌H_{2}{\mathrm{PO}}_{4}^{-}+H^{+}$$

6 $$H_{2}{\mathrm{PO}}_{4}^{-}⇌{\mathrm{HPO}}_{4}^{2-}+H^{+}$$

7 $${\mathrm{HPO}}_{4}^{2-}⇌{\mathrm{PO}}_{4}^{3-}+H^{+}$$

with intrinsic macroscopic acidity constants $pK_{A}^{j}:pK_{A}^{1}=2.16$ , $pK_{A}^{2}=7.21$ , and $pK_{A}^{3}=12.32$ .^51,52^ The populations of the different protonation states are coupled and therefore the ionization equilibria are not independent. Nevertheless, within the HH approximation employed here, phosphate is treated as an idealized reference system in which the release of each proton, indexed by *j* = 1, 2, 3, is represented by an independent ionization event. This approximation enables a treatment formally analogous to the monoprotic case. Accordingly, α_*j*_ denotes the probability that the *j*-th proton has been released and is calculated using eq 3. The average charge of phosphate is then given by

8 $$Q_{\mathrm{phos}}=-e\overset{3}{\underset{j=1}{\sum }}\alpha_{j}$$

## 3 Methods

### 3.1 Constant pH Molecular Simulation

#### 3.1.1 Molecular Model

The molecular model used in this work consists of a single G2 PAMAM dendrimer in an aqueous solution containing sodium, chloride, and phosphate ions. The model explicitly includes electrostatic interactions, steric repulsion, and acid−base equilibria, while neglecting phosphate-specific short-range interactions such as hydrogen bonding, hydration effects, dispersion interactions, and chemical details of ion pairing. Consequently, phosphate accumulation in this model should be interpreted as arising from electrostatics and charge regulation rather than from chemically specific phosphate−PAMAM binding. This minimal representation is useful for isolating the contribution of multivalent titratable ions to the effective charge of cationic dendrimers. Additional technical details are provided in the Supporting Information (cf. Section S1.1).

The dendrimer is constructed using three different bead types representing primary amines, tertiary amines, and neutral groups (Figure 1). Primary and tertiary amine beads are centered on terminal and branching nitrogen atoms, respectively, whereas neutral beads represent the remaining non-ionizable moieties. Branching points are connected by chains of six neutral beads, while the two central amine beads are connected by a shorter segment containing two neutral beads. All beads are linked through harmonic bonds with equilibrium length *r*_0_ = 0.15 nm and are assigned an effective diameter *d*_bead_ ≃ *r*_0_.

The sodium, chloride and phosphate ions in solution are each represented by a single sphere. Monovalent ions are assigned the same diameter as PAMAM beads, whereas phosphate is modeled with a diameter *d*_phos_ = 0.43 nm, consistent with reported ionic and crystal radii.^53,54^ The phosphate charge varies between 0 and −3*e*, depending on its protonation state, while its diameter is assumed to remain constant.

#### 3.1.2 Simulation Method

The simulations in this work employed a hybrid scheme that combines Langevin Dynamics (LD) with constant-pH Monte Carlo (cpH) sampling.^48^ Within each simulation cycle, configurational sampling is performed by integrating the LD equations of motion (Section S1.2), while the acid−base equilibria are sampled via cpH titration trial moves. Quantities of interest are recorded after each simulation cycle for subsequent postprocessing and statistical analysis.

The cpH titration moves modify the protonation states of amine and phosphate groups according to the acid−base reactions defined in Section 2. To maintain global electroneutrality, each protonation or deprotonation event is accompanied by the insertion or deletion of a monovalent cation. These trial moves are accepted according to the constant-pH statistical ensemble,^48^ with acceptance probabilities that depend on the solution pH, the intrinsic p*K*_A_ values, and the local electrostatic environment of the titratable group. The intrinsic p*K*_A_ values used in the simulations are those reported in Section 2; the primary amine value $\left(pK_{A}^{I}=9.15\right)$ differs only slightly from the value reported by Čakara et al. $\left(pK_{A}^{I}=9.0\right)$ .^50^ Following the “Addition of a Cation” procedure described in ref 55, the simulations are corrected for the excess chemical potential of the neutralizing monovalent cation, which is estimated using the Davies equation. A detailed description of the simulation method and the corresponding acceptance probabilities is provided in the Supporting Information (Section S1.3).

The direct inputs of the simulations are the solution pH, the concentrations of phosphate and background salt ions, and the intrinsic p*K*_A_ values of all titratable groups. The simulations yield equilibrium properties, which are characterized using the observables defined in Section 3.1.4.

#### 3.1.3 Computational Details

All simulations were performed using the ESPResSo simulation package (v4.2.2).^56^ The coarse-grained molecular model and simulation setup were generated using pyMBE (v1.0.0).^57^ The simulation box length was set to *L* ≈ 10.85 nm, corresponding to a PAMAM concentration of 1.3 mM and an amine concentration of 39 mM. Although the PAMAM concentration used in the simulations is approximately three times lower than that employed in the potentiometric titrations, both conditions remain within the dilute regime. Therefore, this difference is not expected to significantly affect the measured equilibrium properties, while substantially reducing the computational cost of the simulations. The concentration of the NaCl background electrolyte was fixed at 100 mM, while the phosphate concentration was varied between 9.1 mM and 100 mM. For the chosen box size, this corresponds to 77 background pairs of salt ions and between 7 and 77 phosphate ions, initially in a fully protonated state.

These conditions should be regarded as model phosphate-buffer solutions rather than exact representations of standard PBS. Standard PBS typically contains approximately 10 mM phosphate, 150 mM monovalent salt, and a pH close to 7.4. In the present simulations, the lowest phosphate concentration studied (*C*_phos_ = 9.1 mM) and pH values around 7.4 approach PBS conditions, whereas the NaCl concentration is fixed at 100 mM. Furthermore, because phosphate is a titratable multivalent ion, the ionic strength of the solution is coupled to both *C*_phos_ and pH through phosphate ionization. Consequently, the objective of the simulations is not to reproduce a particular PBS composition, but rather to isolate and quantify the role of phosphate concentration and charge regulation under well-controlled buffer-like conditions.

Each simulation comprised 2.5 × 10^5^ cycles. Each cycle consisted of 1000 LD integration steps, followed by approximately 100 cpH titration attempts. The LD time step and damping constant were set to Δ*t* = 0.005τ and γ = 10 τ^−1^, respectively, where $\tau =\sigma \sqrt{\frac{m}{\varepsilon }}$ . The energy scale was defined as ε = *k*_B_ *T*, with *k*_B_ denoting the Boltzmann constant and *T* = 298.15 K the temperature. The length scale was defined as σ = *d*_bead_. The particle mass *m* was arbitrarily set to unity, as it does not affect the equilibrium properties measured in this study. Typical simulation production runs required approximately 1 day of computation per CPU (Intel Xeon Gold 6242) on the IDUN High Performance Computing cluster.^58^

#### 3.1.4 Measured Quantities

To bridge acid−base equilibria, electrostatic ion correlations, and dendrimer conformational response, we characterize the following equilibrium properties. Ionization equilibria are characterized by the degree of ionization, α, from which the average net charges of PAMAM and phosphate are calculated using eqs 4 and 8, respectively. The conformational properties of PAMAM are quantified by its radius of gyration.

The spatial distribution of species around the dendrimer is characterized by the radial distribution function *g*_*i*_(*r*), which describes the local number density of species *i* at a distance *r* from the dendrimer center, located between the two central amines, normalized by its bulk density,

9 $$g_{i}\left(r\right)=\frac{1}{\rho_{i}^{\mathrm{bulk},n}}\frac{n_{i}\left(r\right)}{dV}=\frac{\rho_{i}^{n}\left(r\right)}{\rho_{i}^{\mathrm{bulk},n}}$$

Here, *n*_*i*_ denotes the average number of particles of species *i* within a spherical shell of volume d*V* = 4π*r* ^2^d*r*, $\rho_{i}^{n}$ is the corresponding number density and $\rho_{i}^{\mathrm{bulk},n}$ is the bulk number density. In practice, $\rho_{i}^{bulk,n}$ is estimated by averaging $\rho_{i}^{n}\left(r\right)$ over the plateau region of the concentration profile at long distances from the dendrimer center. The charge density profile of species *i* around the dendrimer’s center is defined as

10 $$\rho_{i}^{q}\left(r\right)=q_{i}\left(r\right)\rho_{i}^{n}\left(r\right)$$

where *q*_*i*_(*r*) denotes the average charge at distance *r* from the dendrimer center.

To quantify phosphate accumulation in the vicinity of the dendrimer, we calculate the number of adsorbed phosphate ions,

11 $$\Gamma_{N}=\int_{0}^{L/2}\left[\rho_{\mathrm{phos}}^{n}\left(r\right)-\rho_{\mathrm{phos}}^{\mathrm{bulk},n}\right]4\pi r^{2}dr$$

and the corresponding charge associated with the excess phosphate ions, given by

12 $$\Gamma_{Q}=\int_{0}^{L/2}q_{\mathrm{phos}}\left(r\right)\left[\rho_{\mathrm{phos}}^{n}\left(r\right)-\rho_{\mathrm{phos}}^{\mathrm{bulk},n}\right]4\pi r^{2}dr$$

Statistical uncertainties are estimated using the block analysis method described in ref 59.

### 3.2 Site Binding Theory

As a complement to the experimental and simulation results, we employed an adapted version of the Site Binding (SB) model originally proposed by Čakara, Kleimann, and Borkovec for branched macromolecules such as PAMAM dendrimers.^50,60^ Within this framework, each ionizable group is represented by a binary state variable *s*_*i*_, where *s*_*i*_ = 1 corresponds to a protonated site and *s*_*i*_ = 0 to a deprotonated site. A complete protonation configuration, or *microstate*, is thus defined by the set {*s*_*i*_}.

The free energy of a given microstate, measured relative to the fully deprotonated molecule, is expressed as

13 $$\frac{\beta F\left(\left\{s_{i}\right\}\right)}{\ln10}=-\underset{i}{\sum }pK_{A,i}s_{i}+\frac{1}{2}\underset{i,j}{\sum }\varepsilon_{ij}s_{i}s_{j}$$

where p*K*_A,*i*_ denotes the intrinsic acidity constant of site *i*, β = 1/(*k*_B_ *T*) is the inverse thermal energy, and *ε*_*ij*_ quantifies the interaction between protonated sites *i* and *j*. Positive values of *ε*_*ij*_ correspond to repulsive interactions that penalize the simultaneous protonation of neighboring sites. Formally, this model is equivalent to an Ising Hamiltonian, in which protonated and deprotonated groups are represented as interacting two-state variables.

The corresponding partition function is given by

14 $$\Xi =\underset{\left\{s_{i}\right\}}{\sum }a_{H}^{n}e^{-\beta F(\{s_{i}\})}$$

where the sum runs over all possible protonation microstates, *a*_H_ is the proton activity, and *n* = ∑_*i*_ *s*_*i*_ denotes the total number of bound protons in a given microstate. Knowledge of Ξ allows the calculation of macroscopic observables such as the average degree of protonation, macrostate populations, and effective ionization constants. For a polybasic molecule such as PAMAM, the average net charge can be obtained from the partition function as

15 $$Q_{\mathrm{PAMAM}}=-\frac{e}{\ln\left(10\right)}\frac{\partial \ln\Xi }{\partial \mathrm{pH}}$$

In its original formulation, the SB model developed by Borkovec and coworkers for PAMAM dendrimers employed six fitting parameters: distinct intrinsic p*K*_A_ values for primary amines, outer tertiary amines, and the two central tertiary amines, along with three separate nearest-neighbor interaction parameters.^50,60^ This framework successfully reproduced potentiometric titration data of PAMAM and poly­(propyleneimine) dendrimers across different generations and ionic strengths.

In the present work, we employ a simplified version of the model involving only three fitting parameters: an intrinsic $pK_{A}^{I}$ for primary amines, a single intrinsic $pK_{A}^{\mathrm{III}}$ for tertiary amines, and one nearest-neighbor interaction parameter *ε*. Despite its reduced complexity, this minimal representation provides a satisfactory description of the PAMAM titration curves obtained from both potentiometric experiments and constant-pH simulations, as discussed in Section 4.1.

### 3.3 Potentiometric Titration

Potentiometric titrations were performed to benchmark the individual ionization behavior of G2 PAMAM dendrimers and phosphate in aqueous solution. For PAMAM, 18 mg of G2 dendrimers with ethylenediamine cores (Sigma-Aldrich/Merck) were dialyzed against Milli-Q water, freeze-dried, and subsequently dissolved in 1.7 mL of standardized 0.1 M HCl, corresponding to a PAMAM concentration of 3.3 mM. The solution was titrated with standardized 0.1 M NaOH at a temperature of 296.15 K. Phosphate titrations were conducted using 3.4 mg of Na_3_PO_4_ (96% purity, Sigma-Aldrich), which was dissolved in 2.0 mL of standardized 0.1 M NaOH under an inert atmosphere, corresponding to a Na_3_PO_4_ concentration of 9.95 mM, and titrated with standardized 1 M HCl at a temperature of 293.15 K. All titrations were carried out under controlled temperature conditions using automated titration systems equipped with glass pH electrodes and magnetic stirring. Blank titrations were performed under identical conditions to account for background contributions. Standardized acid and base solutions were prepared from certified ampules, and all samples were prepared using ultrapure water. Further details regarding sample preparation, titration protocols, instrumentation, and data analysis are provided in the Supporting Information (Section S2).

## 4 Results and Discussion

### 4.1 Independent Ionization of PAMAM and Phosphate in Dilute Solution

The ionization behavior of phosphate and PAMAM in dilute solution, where intermolecular interactions are negligible, provides a necessary reference for interpreting more complex conditions in which both species coexist and their ionization equilibria may become coupled.

As shown in Figure 2A, the net charge of PAMAM decreases with increasing pH in a characteristic two-step manner. The first transition, occurring at acidic pH (around p*K*_A_ = 6.0), is associated with the ionization of tertiary amines located in the dendrimer core. In this regime, the constant-pH simulation results (cpH, blue circles), potentiometric titration experiments (Exp., diamonds and crosses) and the Site Binding theory (SB, solid purple line) fall below the Henderson−Hasselbalch (HH) baseline (eqs 3–4, black continuous line), indicating that intramolecular electrostatic repulsions strongly suppress the ionization of the tertiary amines.

The second transition, observed at basic pH (around p*K*_A_ = 9.15), corresponds to the ionization of primary amines at the dendrimer periphery. In this regime, the measured charge deviates only slightly from the HH prediction, with differences comparable to those expected for a monoprotic base in the presence of monovalent salt.^55,62^ This weaker deviation from ideality can be attributed to two factors. First, the tertiary amines are already largely deprotonated at these pH values, reducing the overall dendrimer charge and, consequently, the magnitude of intramolecular electrostatic repulsion. Second, the primary amines are more spatially separated than the tertiary amines, which further weakens their electrostatic coupling. Consistent with this interpretation, higher-generation PAMAM dendrimers, which exhibit a higher surface density of primary amines, show larger deviations from ideal ionization behavior.^50^ Overall, these results indicate that intramolecular electrostatic interactions play a more prominent role in determining the ionization behavior of G2 PAMAM at low pH, where the dendrimer carries a high net positive charge.

Across most of the pH range, we observe very good, often nearly quantitative, agreement between the cpH simulations and the PAMAM net charge measured in potentiometric titration experiments, both in this work (orange crosses in Figure 2) and as reported in the literature^50^ (green diamonds in the same figure). Small deviations between simulations and our experiments are observed only around pH ∼6, which may reflect specific interactions not captured by the coarse-grained model, such as hydrogen bonding or hydrophobic effects. Our potentiometric titration data are also in excellent agreement with the results of Čakara et al.,^50^ although minor differences are apparent at both low and high pH values. These discrepancies may arise from the different background electrolytes used in the two studies: Čakara et al. employed 0.1 M KCl, whereas we used 0.1 M NaCl. The ionic activity coefficients of Na^+^ and K^+^ differ by approximately 0.04 units in a 1:1 0.1 M electrolyte solution,^63^ which could account for the small but systematic differences between the two data sets. Overall, these results indicate that the proposed coarse-grained model provides a reliable and quantitatively accurate description of the ionization behavior of PAMAM in dilute solutions.

The Site Binding (SB) model introduced in Section 3.2 reproduces the experimental titration curve remarkably well (golden solid line). Noticeable deviations between the SB model and the experimental data are observed only at basic pH values, corresponding to the ionization of the primary amines. Fitting the model to the potentiometric data yields intrinsic acidity constants of $pK_{A}^{I}=8.96$ for primary amines and $pK_{A}^{\mathrm{III}}=6.31$ for tertiary amines, together with a nearest-neighbor interaction parameter *ε* = 0.38. Despite reducing the number of fitting parameters from six in the original model proposed by Čakara et al.^50^ to only three, the fitted parameters remain in reasonable agreement with the original values ( $pK_{A}^{I}=9.00$ , $pK_{A}^{\mathrm{III}}=6.00$ and *ε* = 0.15). This result indicates that the additional parameters introduced in the original formulation provide only minor corrections for G2 PAMAM under the conditions considered here. In particular, Čakara et al. included an additional lateral interaction parameter, *ε*″, accounting for interactions between primary amines, which may explain the residual discrepancies observed at basic pH. We further fitted the cpH simulation results to the SB model and obtained similarly excellent agreement between theory and simulations (cf. Section S5 in the Supporting Information). Notably, the SB model quantitatively reproduces the individual contributions of primary and tertiary amines to the total PAMAM charge without requiring additional fitting parameters. Altogether, the close agreement between SB theory, simulations, and experiments suggests that the protonation free energy of G2 PAMAM is dominated by nearest-neighbor electrostatic interactions between amine groups, while longer-range and higher-order contributions play only a secondary role.

For phosphate (Figure 2B), the absolute net charge increases with increasing pH, displaying the opposite pH dependence to that observed for PAMAM. The ionization occurs through three distinct steps, corresponding to the successive deprotonation reactions described in eqs 5–7.

Deviations from the ideal HH prediction are observed in both the cpH simulations and the potentiometric titration data, particularly near each ionization transition. Since these measurements correspond to the individual titration of phosphate in the absence of PAMAM, the deviations arise from electrostatic interactions with the background electrolyte and neighboring phosphate ions in solution. Importantly, the magnitude of the deviation increases with pH as phosphate progressively changes from a monovalent to a trivalent species, thereby increasing both the electrostatic self-interactions between phosphate ions and the ionic strength of the solution. This effect is particularly pronounced for the third ionization step, where the transition from divalent to trivalent phosphate produces the largest increase in ionic strength and, consequently, the strongest departure from ideal HH behavior. For reference, we quantified the pH-dependent ionic strength of the phosphate solution, which shows an appreciable phosphate contribution, especially at basic pH (Figure S5A in the Supporting Information). Across the intermediate pH range accessible to potentiometric titration, ranging from 3 to 11 pH units, we observe excellent quantitative agreement between the cpH simulations and our experimental data.

To further assess the validity of the cpH simulations at extreme pH values, corresponding to the first and third phosphate ionization steps, we compared the effective acidity constants obtained from the simulations with literature data. From the simulation results, we estimate effective acidity constants of $pK_{A}^{\mathrm{eff},1}=1.88$ , $pK_{A}^{\mathrm{eff},2}=6.60$ , and $pK_{A}^{\mathrm{eff},3}=11.20$ by fitting the phosphate titration curves to the HH model (eqs 3–8; see Supporting Information, Section S6). These values are in good agreement with the expected experimental effective acidity constants for phosphate in a 0.1 M NaCl solution, namely $pK_{A}^{\mathrm{eff},1}\approx 1.91$ , $pK_{A}^{\mathrm{eff},2}\approx 6.74$ , and $pK_{A}^{\mathrm{eff},3}\approx 11.63$ . The reference values were obtained using the semiempirical formulation proposed by Daniele et al.,^61^ which was parametrized to reproduce phosphate effective acidity constants over a broad range of electrolyte compositions, ionic strengths, and temperatures (see Supporting Information, Section S4).

For comparison, these experimental $pK_{A}^{\mathrm{eff}}$ values were used to compute an HH prediction incorporating electrolyte effects (effective HH model, green solid line in Figure 2B). Notably, this effective HH description reproduces the potentiometric titration data very accurately. The cpH simulations also reproduce the effective HH behavior very well, particularly in the pH range associated with the first and second deprotonation steps (eqs 5 and 6). At highly basic pH values, corresponding to the third deprotonation step (eq 7), the simulations exhibit a slight but systematic overestimation of the absolute phosphate charge. This discrepancy likely originates from the Davies approximation used to estimate the activity coefficient of the neutralizing counterion within the cpH scheme (cf. Section S1.3 in the Supporting Information), which is known to become less accurate for multivalent ions.^55,62^ Although this limitation could, in principle, be overcome by explicitly calculating the chemical potential of the neutralizing counterions in each simulation, the present results indicate that the Davies approximation provides a satisfactory compromise between accuracy and computational efficiency under the conditions investigated here.

Overall, these findings demonstrate that the coarse-grained model captures the ionization behavior of both PAMAM and phosphate in dilute solution with good accuracy, providing a robust foundation for investigating their coupled ionization in more complex environments.

**Figure 2:**
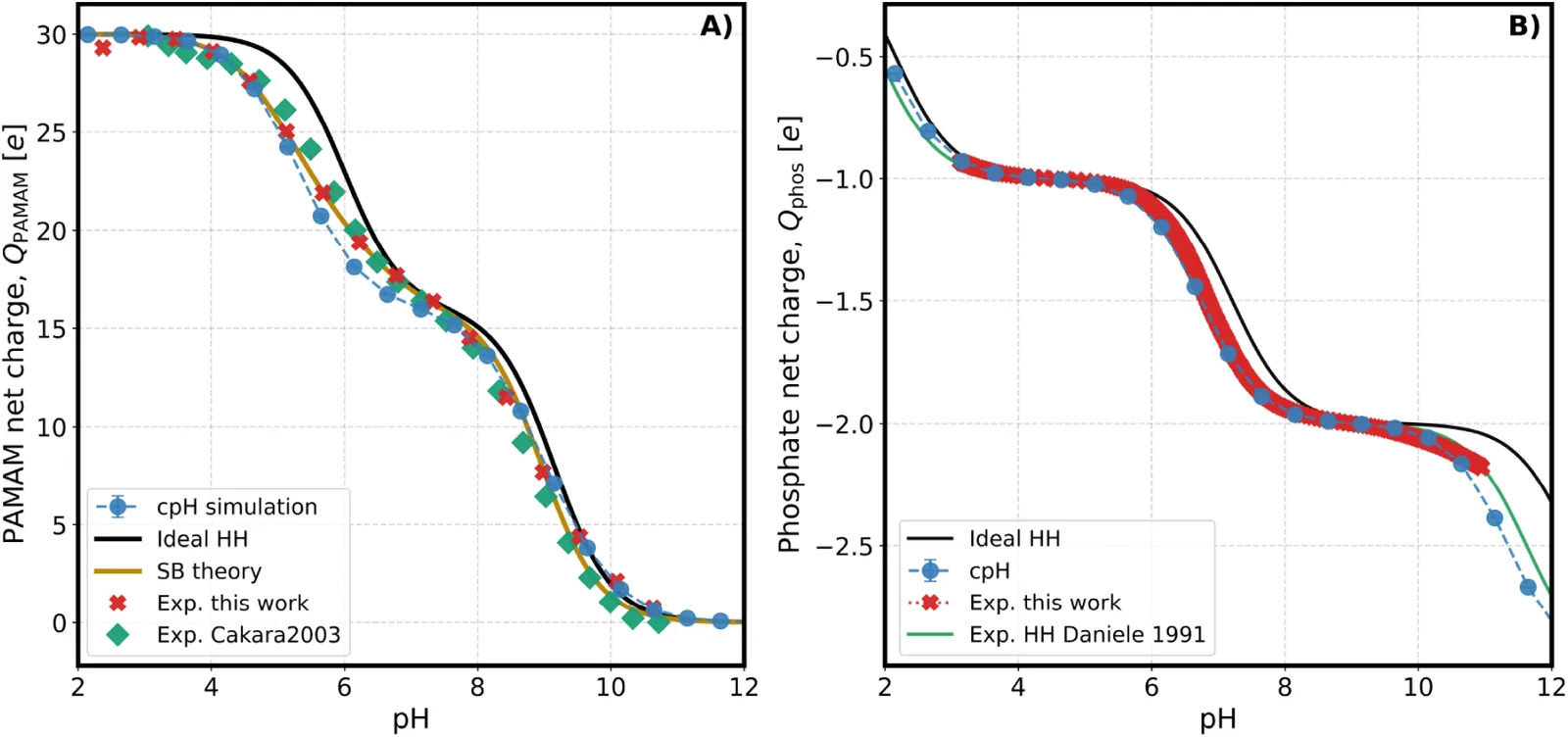
Panel A: Net charge of PAMAM as a function of pH in aqueous 0.1 M NaCl solution. Constant-pH simulation results (blue circles) and potentiometric titration data, obtained in this work (red crosses) and reported by Čakara et al.^50^ (green diamonds), are compared with the Henderson−Hasselbalch (HH) prediction calculated using the input (bare) p*K*_A_ values (eqs 3–4, black solid line). The best fit to the titration data measured in this work, obtained using the Site Binding (SB) model (cf. Section 3.2), is shown as a golden solid line. Panel B: Net charge of phosphate as a function of pH in aqueous 0.1 M NaCl solution. Constant-pH simulation results (blue circles, *C*_phos_ = 9.1 mM) are compared with potentiometric titration data measured in this work (red crosses, *C*_phos_ = 9.9 mM). HH predictions are also shown (eqs 3–8) calculated using (i) the input (bare) p*K*_A_ values (black solid line) and (ii) effective acidity constants $pK_{A}^{\mathrm{eff}}$ estimated for phosphate in approximately 0.1 M NaCl using the semiempirical model of Daniele et al.^61^ (green solid line), as described in Section S4 of the Supporting Information.

### 4.2 Coupled Ionization of PAMAM and Phosphate in Buffer Solution

The results presented thus far describe the ionization behavior of PAMAM and phosphate in the absence of mutual interactions. We now consider the case of interest, in which both species coexist in solution. Under these conditions, electrostatic interactions can couple their ionization equilibria, potentially leading to mutual charge regulation.^64^ Such coupling may also promote the accumulation of phosphate ions near the dendrimer, thereby modifying local ion distributions as well as the structural properties of PAMAM. In the following, we examine these effects in detail.

Experimentally, disentangling the individual charge contributions of PAMAM and phosphate in potentiometric titration is highly challenging. The primary observable in such experiments is the pH as a function of added titrant volume, which can be related to the total analyte charge (cf. Section S2.2). However, because both PAMAM and phosphate ionize over a similar pH range, separating their individual contributions requires additional assumptions. For example, one may fix the charge of one component based on a theoretical model or assume that its ionization behavior is not affected by the other components, effectively neglecting mutual charge regulation effects. Consistent with this limitation, we show in Section S2.3 of the Supporting Information that the simulated total titration curve of the mixed PAMAM−phosphate system is nearly indistinguishable from the additive combination of the separately simulated PAMAM and phosphate titration curves, with deviations below approximately 0.5*e* over the full pH range. This indicates that local phosphate accumulation and local charge regulation are largely masked in the total titration charge and would be difficult to resolve reliably by conventional potentiometric titration alone. In contrast, cpH molecular simulations provide direct access to the charge state of each species independently, enabling a detailed and unambiguous characterization of their coupled ionization behavior. For this reason, in the following, we focus exclusively on results obtained from cpH molecular simulations.

#### 4.2.1 Mutual Charge Regulation between PAMAM and Phosphate

The cpH simulations (markers in Figure 3A) indicate that the presence of phosphate has only a minor effect on the PAMAM net charge. Specifically, a slight increase in the net charge is observed with increasing phosphate concentration, bringing the ionization behavior closer to the ideal HH baseline (black line). This effect is most pronounced at intermediate pH values (approximately 5−9), and becomes negligible at both low and high pH. This trend can be attributed to favorable electrostatic interactions between the positively charged amine groups of PAMAM and negatively charged phosphate species, which stabilize the protonated states of the dendrimer.

Kéri et al.^30^ reported NMR measurements of $pK_{A}^{\mathrm{eff}}$ values of amine groups in generation 5 (G5) PAMAM dendrimers in the absence and presence of 100 mM phosphate. They observed a shift of the effective p*K*_A_ values toward higher pH by approximately 0.8 units in the presence of phosphates. This observation is qualitatively consistent with our results, which likewise indicate that phosphate stabilizes the protonated state of PAMAM. However, the magnitude of the effect observed by Kéri et al. is considerably larger than the predicted here. This difference is likely related to the substantially larger number of ionizable amine groups in G5 PAMAM (254 versus 30 in G2), which generates stronger electrostatic fields and is therefore expected to amplify charge-regulation effects.^64,65^

In contrast to PAMAM, phosphate exhibits clear shifts in its net charge relative to the isolated case (blue circles), as shown in Figure 3B. In all cases, the absolute net charge of phosphate increases in the presence of PAMAM, further deviating from the ideal HH baseline (black line). This effect is particularly pronounced in the pH regions corresponding to the second and third deprotonation steps (eqs 6 and 7). This behavior complements that observed for PAMAM: favorable electrostatic interactions with the positively charged dendrimer stabilize the deprotonated states of phosphate, providing clear evidence of mutual charge regulation. A similar behavior was reported by Kéri et al.,^30^ who observed enhanced phosphate ionization in the presence of G5 PAMAM dendrimers.

Returning to Figure 3B, it is interesting to note that the magnitude of the observed effect increases with phosphate concentration. Because phosphate is polyprotic, increasing *C*_phos_ also changes the pH-dependent ionic strength of the solution, specially at high *C*_phos_ and basic pH, as shown in Figure S5B in the Supporting Information. Accordingly, the concentration-dependent trends reported here should be interpreted as the combined effect of phosphate availability, charge regulation, and changes in ionic strength, rather than as purely phosphate-specific effects. In addition, the net phosphate charge shown in Figure 3B represents an average over phosphate ions both in the vicinity of PAMAM and in the bulk solution. As a result, the direct contribution of local charge regulation near the dendrimer is partially masked in the global average charge.

**Figure 3:**
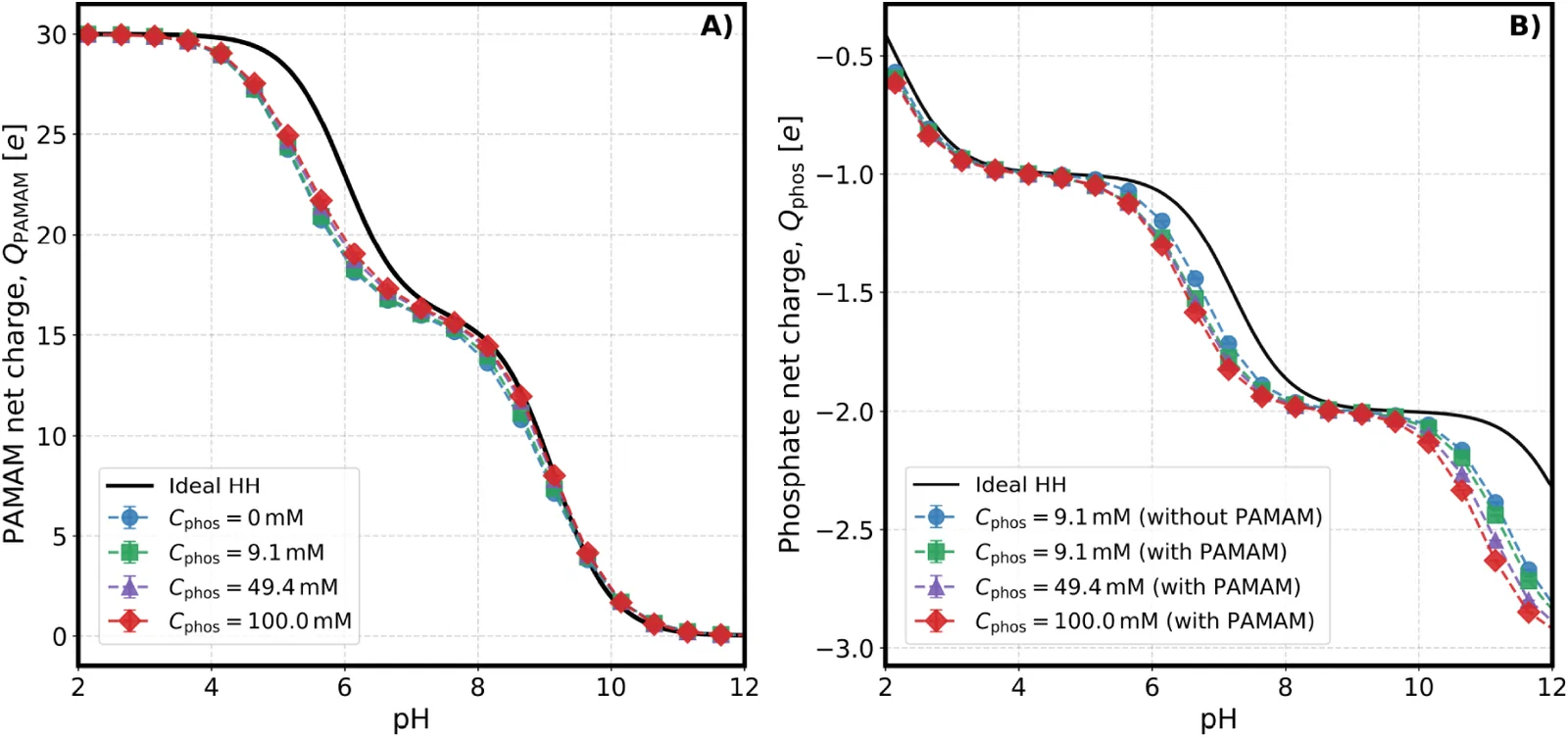
Panel A: Net charge of PAMAM as a function of pH at different phosphate concentrations *C*_phos_, with 0.1 M NaCl as background electrolyte. Results from constant-pH simulations (cpH, symbols) are compared with the Henderson−Hasselbalch (HH) prediction (black solid line), calculated using eqs 3–4. Panel B: Net charge of phosphate in the presence of PAMAM at different phosphate concentrations *C*_phos_, with 0.1 M NaCl as background electrolyte. For reference, constant-pH simulation results for the individual ionization of phosphate at *C*_phos_ = 9.1 mM, i.e., in the absence of PAMAM, are also shown (blue circles). The HH prediction is included as an ideal baseline, calculated using eqs 3–8.

To quantify how the local electrostatic environment generated by PAMAM modulates the ionization of nearby phosphate ions, we analyze the average net charge of phosphate as a function of the distance from the center of the dendrimer (Figure 4A). We observe a monotonic increase in the absolute charge of phosphate ions as they approach PAMAM, with differences of up to ∼0.6 *e* between bulk ions and those in proximity to the dendrimer at pH = 2.0 and pH = 5.0. This effect is consistent across phosphate concentrations, with only minor variations between *C*_phos_ values, typically within statistical uncertainty, indicating that the ionic strength and phosphate−phosphate interactions play a negligible role in this regime. Little or no charge regulation of phosphate ions near PAMAM is observed at pH = 8.0 and pH = 11.0, despite the latter being close to the third effective acidity constant, $pK_{A}^{\mathrm{eff}}$ , of phosphate. This is because at those pH values PAMAM is nearly neutral. A noticeable concentration dependence is observed only at pH = 11, where phosphate is predominantly trivalent and therefore contributes significantly to the ionic strength of the solution, enhancing electrostatic screening effects.

The pH values where a molecule is more susceptible to regulate its charge can be identified by the maxima on its charge regulation capacity defined as the derivative of the charge with respect to pH^66,67^

16 $$C=-\frac{dQ}{d\mathrm{pH}}=N\;\ln\left(10\right)\left(\left\langle Q^{2}\right\rangle -{\langle Q\rangle }^{2}\right)$$

where *N* is the number of titratable groups. PAMAM exhibits two maxima in C at pH ≈ 5 and pH ≈ 9, while phosphate shows three maxima at pH ≈ 2, pH ≈ 7 and pH ≈ 11, as shown in Figure 4B for *C*_phos_ = 100 mM. In both cases, the maxima occur close to the corresponding effective acidity constants, $pK_{A}^{\mathrm{eff}}$ . The positions of these maxima depend only weakly on *C*_phos_ (see Supporting Information, Section S8). The weak charge regulation response of PAMAM occurs primarily at pH values close to the maxima in $C_{\mathrm{PAMAM}}$ (cf. Figure 3A), where the dendrimer is most sensitive to changes in its electrostatic environment. The charge regulation capacitance also provides additional insight into the phosphate behavior shown in Figure 4A. At pH = 2, phosphate ions near PAMAM exhibit significant charge regulation, consistent with the first maximum in $C_{\mathrm{phos}}$ . A similarly strong response is observed at pH = 5, despite the more moderate value of $C_{\mathrm{phos}}$ , indicating that PAMAM remains sufficiently charged to induce phosphate charge regulation. In contrast, phosphate ions show little charge regulation at pH = 8, even though $C_{\mathrm{phos}}$ is comparable to that at pH = 5, suggesting that the lower net charge of PAMAM at this pH is no longer sufficient to significantly perturb phosphate ionization. Finally, at pH = 11, close to the third maximum in $C_{\mathrm{phos}}$ , phosphate exhibits a weak response to phosphate concentration but essentially no response to PAMAM, which is nearly neutral under these conditions.

Altogether, these findings reveal an asymmetric mutual charge regulation between PAMAM and phosphate: while the presence of phosphate has only a minor impact on the ionization of PAMAM, phosphate ions in the vicinity of the dendrimer can exhibit a substantial increase in their absolute net charge.

**Figure 4:**
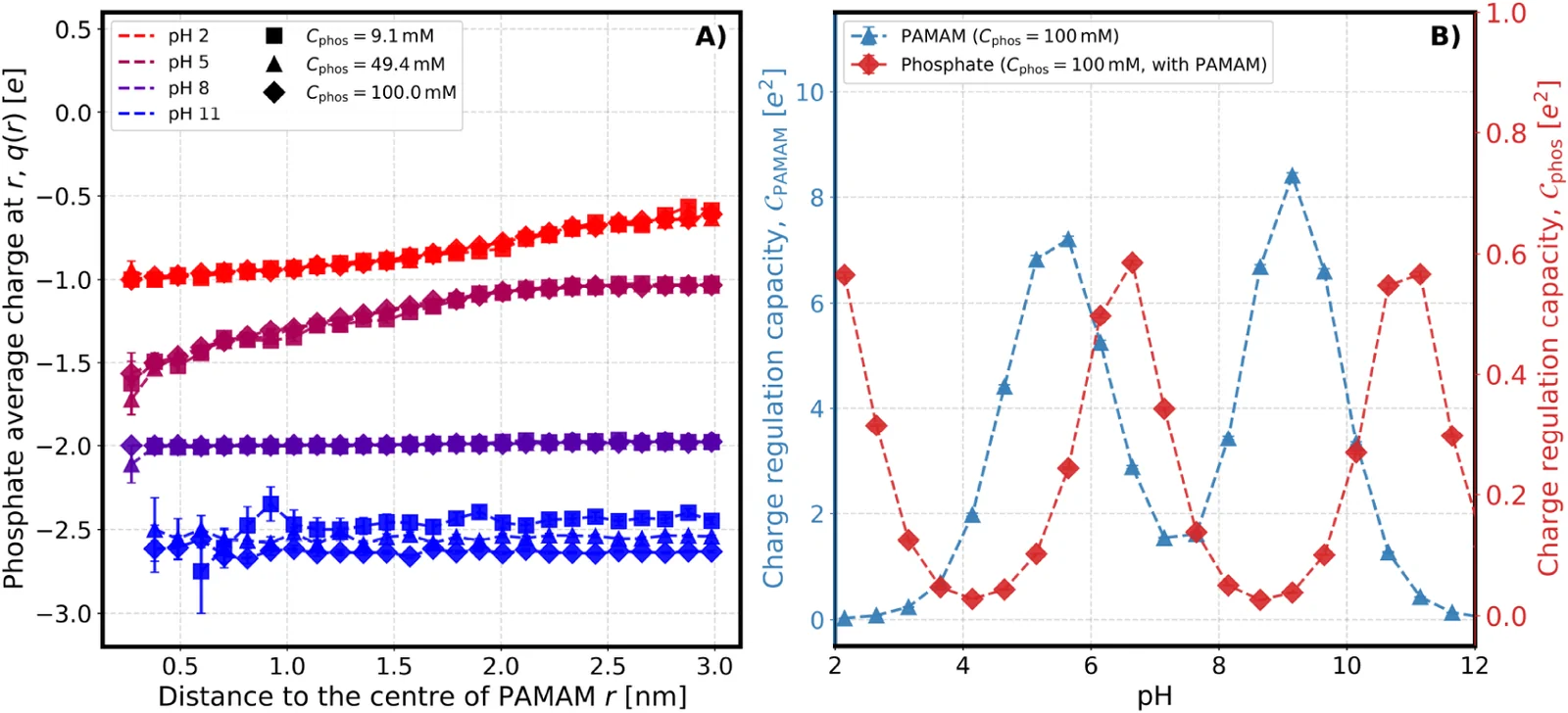
Panel A: Average phosphate charge as a function of distance from the center of the PAMAM dendrimer, *r*, for selected pH values (colors) and phosphate concentrations *C*_phos_ (symbols). Panel B: Charge regulation capacity C of PAMAM (blue triangles, left *y*-axis) and phosphate (red diamonds, right *y*-axis) at phosphate concentration *C*_phos_ = 100 mM.

#### 4.2.2 Phosphate Accumulation onto the PAMAM

Having established the presence of asymmetric mutual charge regulation, we now examine how these electrostatic effects translate into the spatial organization and accumulation of phosphate ions around PAMAM.

The radial distribution functions *g*(*r*) of the phosphate ions around the center of the dendrimer reveal a pronounced accumulation of phosphate in the vicinity of PAMAM at low and intermediate pH values, as shown in Figure 5A for *C*_phos_ = 100 mM and for the lower phosphate concentrations in Section S9 in the Supporting Information. In contrast, at high pH, *g*(*r*) indicates a depletion of phosphate near the dendrimer, despite phosphate being nearly fully deprotonated (trivalent) under these conditions. This behavior reflects the weak net charge of PAMAM at high pH, which reduces electrostatic attraction, combined with steric exclusion that limits phosphate access to the dendrimer interior. As a result, the accumulation exhibits a nonmonotonic dependence on pH, with *g*(*r*) reaching a maximum at intermediate pH values (approximately 4−8). These conditions correspond to a regime in which both PAMAM and phosphate are significantly charged (cf. Figure 3), leading to enhanced electrostatic attraction and increased local accumulation of phosphate ions.

Phosphate accumulation is accompanied by a pronounced regulation of its charge, as shown in Figure 4A. The phosphate charge density, ρ^*q*^_phos_(*r*), shown in Figure 5B, weights the radial distribution function *g*(*r*) by the local phosphate charge *q*(*r*), thereby simultaneously capturing phosphate accumulation and charge regulation effects. Two main trends can be identified. First, at large distances from the dendrimer, ρ^*q*^_phos_(*r*) becomes increasingly negative with increasing pH, reflecting the progressive deprotonation of phosphate ions (cf. Figure 3B). Second, at short distances, ρ^*q*^_phos_(*r*) becomes substantially more negative at low and intermediate pH, consistent with the enhanced accumulation of phosphate ions in the vicinity of the dendrimer. Interestingly, although phosphate is predominantly trivalent at high pH, the local charge density near PAMAM is more negative at low and intermediate pH. This behavior arises because electrostatic attraction strongly confines phosphate ions within the dendrimer environment under these conditions, leading to a high local concentration of mono- and divalent phosphate species. By contrast, at high pH, the weakly charged dendrimer is unable to retain phosphate ions in its vicinity, resulting in local depletion despite the higher individual phosphate charge. Altogether, these results provide clear evidence for the local accumulation of phosphate ions around PAMAM under conditions where electrostatic attraction is strongest, including near-neutral pH values typical of PBS buffers.

**Figure 5:**
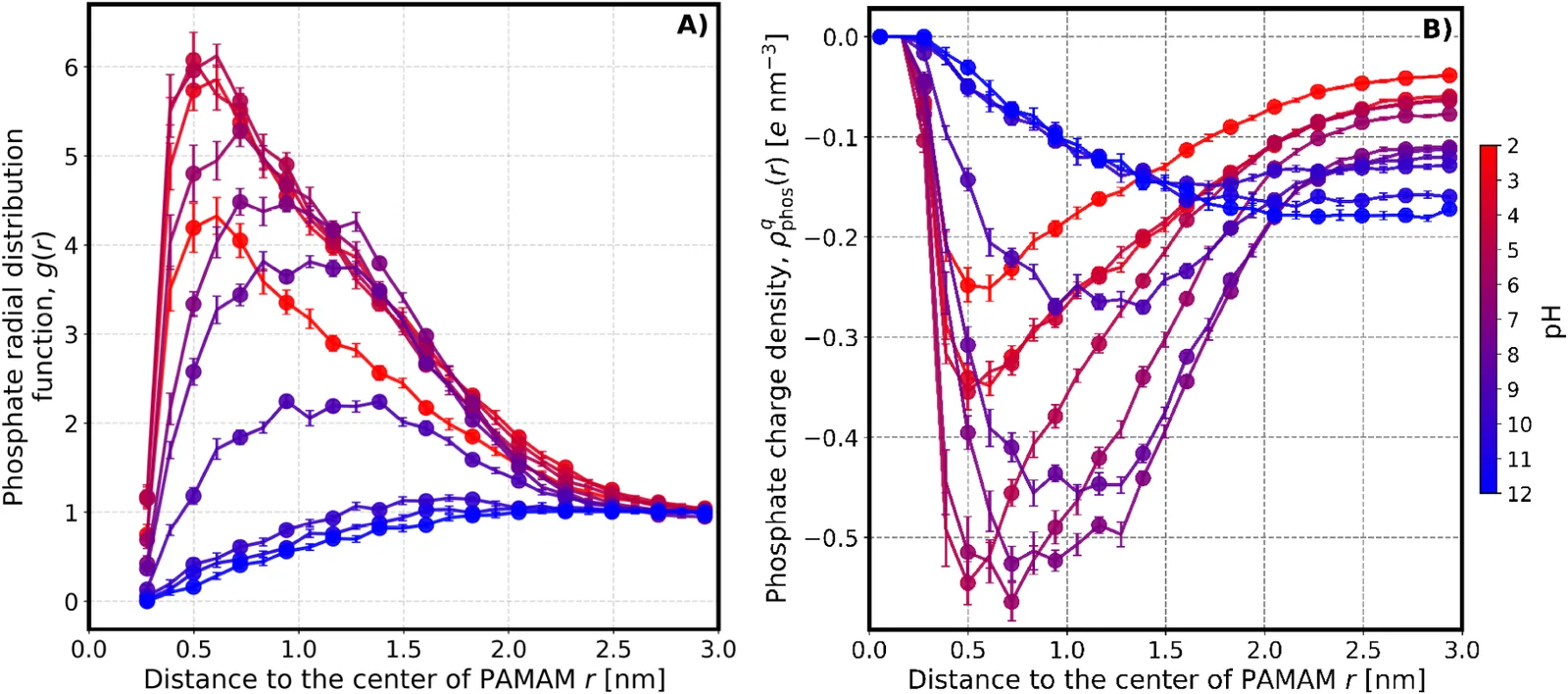
Phosphate spatial distributions around PAMAM at *C*_phos_ = 100 mM for selected pH values. Panel A: radial distribution function *g*(*r*) of phosphate ions around the center of the dendrimer. Panel B: phosphate charge density ρ­(*r*) as a function of the distance to the dendrimer center. The color gradient ranges from pH = 2 (red) to pH = 12 (blue).

In the present coarse-grained model, phosphate accumulation is driven only by electrostatic interactions and acid−base equilibria, since no attractive phosphate-specific short-range interactions are included. Therefore, in the discussion that follows, the term adsorption refers solely to an excess local concentration of phosphate around PAMAM relative to the bulk, and should not be interpreted as evidence of chemically specific binding. The number of phosphate ions adsorbed to PAMAM, Γ_N_, can be quantified from the local and bulk phosphate number densities (eq 11) and is shown in Figure 6A. Positive values of Γ_N_ indicate an accumulation of phosphate ions in the vicinity of PAMAM relative to the bulk, whereas negative values correspond to depletion. A pronounced, nonmonotonic pH dependence is observed, with maximum accumulation at intermediate pH values (approximately 4−8), where both PAMAM and phosphate are significantly charged. At high pH, Γ_N_ decreases sharply and approaches zero or slightly negative values, reflecting reduced electrostatic attraction and the resulting depletion of phosphate near the dendrimer. At low and intermediate pH, Γ_N_ increases with *C*_phos_, reflecting the larger number of phosphate ions available for adsorption.

The adsorbed phosphate fraction, Γ_N_/*N*_phos_ (Figure 6B), provides a normalized measure of phosphate adsorption. At low pH, the adsorbed fraction remains relatively low (approximately 5−10%) and shows little dependence on *C*_phos_. In contrast, at intermediate pH, a clear maximum is observed, reaching approximately 15−20% for *C*_phos_ = 9.1 mM, indicative of particularly significant phosphate adsorption. However, because this lowest-concentration case contains only a small phosphate population in the simulation box, the adsorbed fraction is sensitive to finite-size and sampling effects. Therefore, the precise numerical value of this fraction should be interpreted with caution. As *C*_phos_ increases, this maximum becomes less pronounced, and the adsorbed fraction approaches the values observed at lower pH. At *C*_phos_ = 100 mM, the adsorbed phosphate fraction reaches a plateau at intermediate pH, suggesting that the dendrimer approaches saturation toward phosphate adsorption.

The phosphate accumulation observed in our simulations is qualitatively consistent with the experimental findings of Kéri et al., who used diffusion NMR spectroscopy to investigate phosphate association with G5 PAMAM dendrimers. They reported the presence of a population of phosphate ions that diffuses together with the dendrimers over the pH range 1−7, indicating persistent phosphate association under acidic and near-neutral conditions.^30^ In our simulations, phosphate accumulation is observed over a similarly broad pH range, extending from approximately pH 2 to pH 8 (Figure 6A), with maximum adsorption occurring at intermediate pH where both PAMAM and phosphate are significantly charged. Furthermore, Kéri et al. found that the fraction of associated phosphate ions increases with phosphate concentration at pH 7.^30^ A comparable trend is observed in our simulations, where the number of adsorbed phosphate ions, Γ_*N*_, increases systematically with phosphate concentration (Figure 6A). Although direct quantitative comparison is not possible because of the different dendrimer generations and the different definitions of association employed, the qualitative agreement is noteworthy. In particular, our coarse-grained model reproduces the experimentally observed phosphate association without invoking phosphate-specific short-range interactions, suggesting that electrostatic attraction and mutual charge regulation constitute a robust and generic mechanism underlying phosphate accumulation in PAMAM dendrimers, while phosphate-specific interactions may provide additional quantitative contributions.

**Figure 6:**
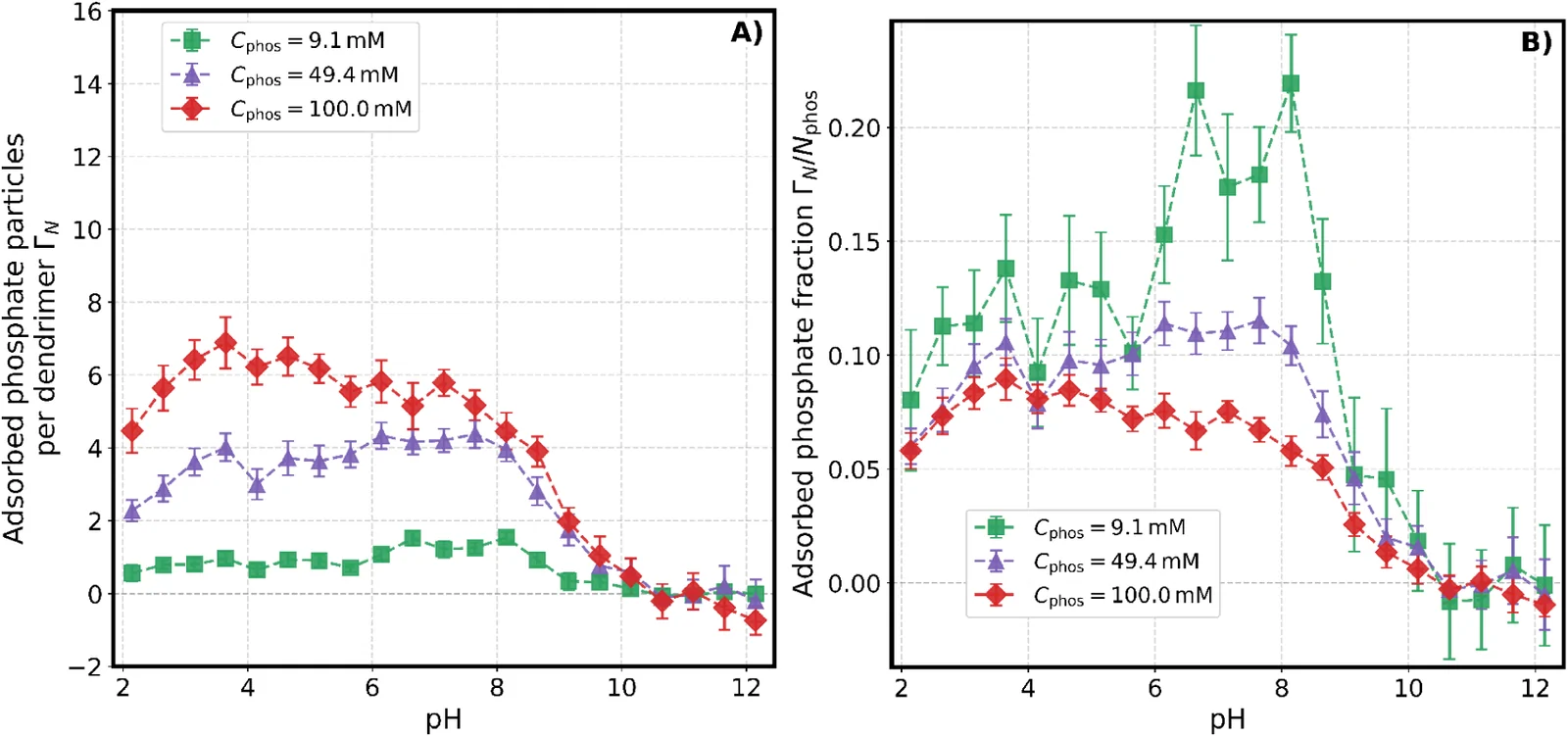
Excess number of phosphate ions adsorbed in PAMAM, Γ_N_ (panel A, cf. eq 11), and the adsorbed phosphate fraction, Γ_N_/*N*_phos_ (panel B), where *N*_phos_ denotes the total number of phosphate ions, shown as a function of pH for different phosphate concentrations, *C*_phos_.

The adsorbed phosphate charge, Γ_*Q*_ (Figure 7A), quantifies the net charge contributed by phosphate ions adsorbed onto the dendrimer. It follows trends similar to those observed for Γ: Γ_*Q*_ is negative due to phosphate accumulation, exhibits a pronounced minimum at intermediate pH where adsorption is strongest, and approaches zero at high pH as phosphate is depleted from the vicinity of PAMAM. The slightly positive values observed at high pH are an artifact of the analysis, reflecting phosphate depletion rather than the presence of positively charged species.

Eventually, the phosphate adsorption results in an effective charge of the dendrimer, which is lower than its bare charge. We define the effective charge of the PAMAM−phosphate complex as

17 $$Q_{\mathrm{PAMAM}}^{\mathrm{eff}}=Q_{\mathrm{PAMAM}}+\Gamma_{Q}$$

As shown in Figure 7B, $Q_{\mathrm{PAMAM}}^{\mathrm{eff}}$ decreases with increasing phosphate concentration and approaches charge neutralization at intermediate pH values (approximately 7−10) for moderate to high *C*_phos_. At *C*_phos_ = 9.1 mM, $Q_{\mathrm{PAMAM}}^{\mathrm{eff}}$ closely follows the charge of independently ionized PAMAM (blue circles) at both low and high pH. However, at intermediate pH, $Q_{\mathrm{PAMAM}}^{\mathrm{eff}}$ remains significantly reduced relative to *Q*_PAMAM_, indicating persistent phosphate adsorption even at the lower concentration. The lowest phosphate concentration considered here, *C*_phos_ = 9.1 mM, together with near-neutral pH values, is close to the phosphate concentration and pH commonly used in PBS buffers, which are widely employed to mimic biological conditions in drug delivery applications involving polycationic carriers, such as PAMAM dendrimers. Within our electrostatic model, the results suggest that multivalent titratable buffer ions can substantially reduce the effective charge of cationic carriers. In real phosphate−PAMAM systems, phosphate-specific interactions such as hydrogen bonding may further enhance this association. In addition, we note that the present results were obtained for a relatively small dendrimer (G2 PAMAM) with a moderate overall charge. For higher-generation dendrimers or more highly charged polycations, the strength of electrostatic interactions, and consequently the extent of charge regulation, is expected to increase significantly. In such systems, stronger local electric fields may further enhance phosphate ionization and promote more pronounced ion accumulation and charge regulation. This suggests that the coupling between multivalent buffer ions and polyelectrolytes identified here may become even more important for larger or more highly charged macromolecular systems.

**Figure 7:**
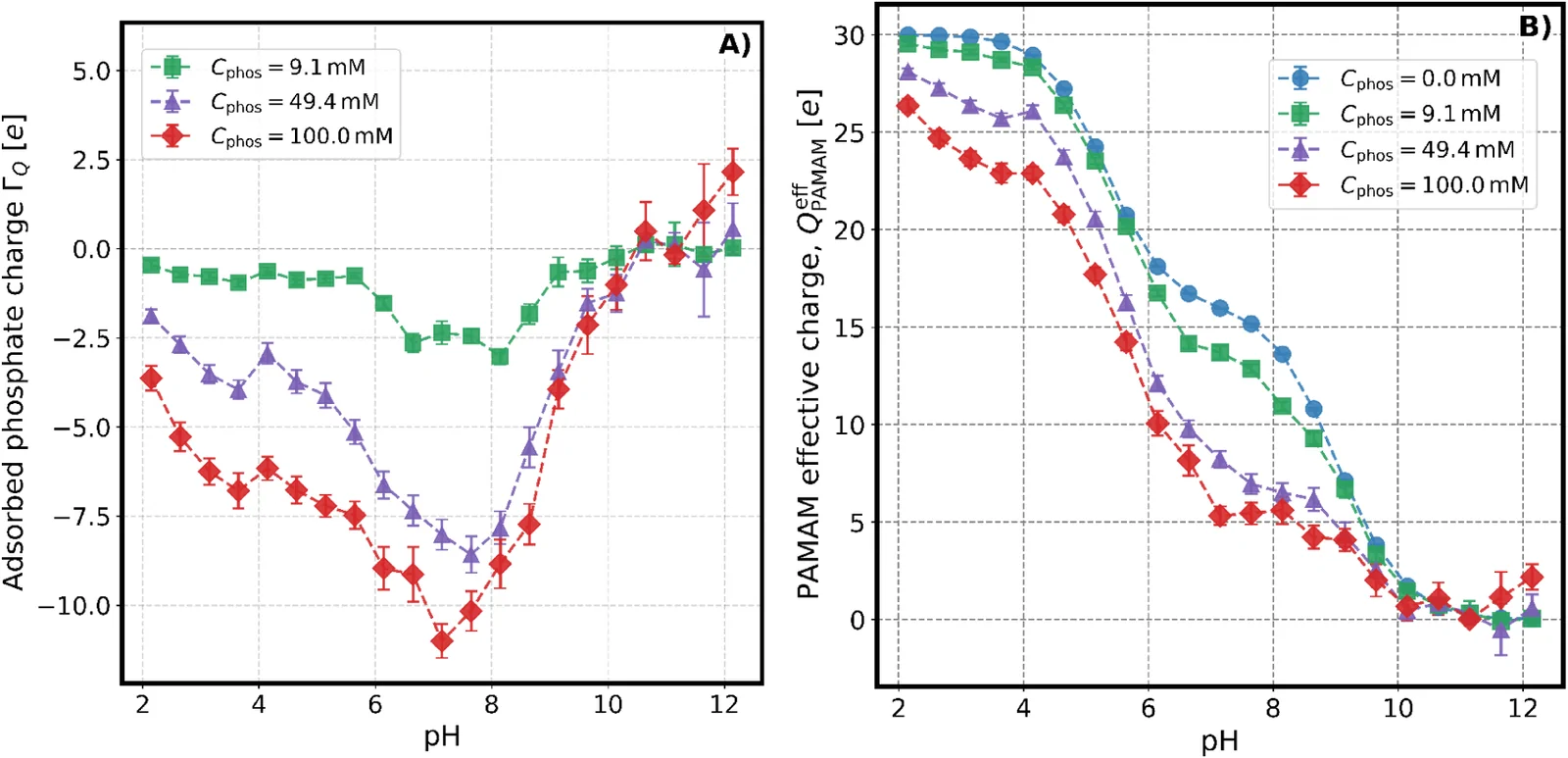
Adsorbed phosphate charge, Γ*_Q_* (panel A, cf. eq 12), and effective charge of the dendrimer−phosphate complex, $Q_{\mathrm{PAMAM}}^{\mathrm{eff}}$ (Panel B, cf. eq 17), as functions of pH for different phosphate concentrations *C*_phos_.

#### 4.2.3 Effect of Phosphate on PAMAM Structure

We assess the effect of phosphate on the structure of PAMAM by analyzing the radius of gyration, *R*_g_ (Figure 8A), and the local concentration of PAMAM monomers, c_mon_(*r*), with respect to the center of the dendrimer (Figure 8B) across a range of pH values and phosphate concentrations *C*_phos_.

The radius of gyration decreases with increasing pH, reflecting the progressive decrease in ionization of PAMAM and the associated reduction in intramolecular electrostatic repulsion. Overall, the influence of *C*_phos_ on *R*_g_ remains modest and becomes noticeable primarily at low and intermediate pH values. At low pH, *R*_g_ increases with *C*_phos_, indicating that the phosphate ions adsorbed onto the dendrimer lead to a small expansion of the dendrimer, likely due to steric constraints. In contrast, at intermediate pH, *R*_g_ decreases with increasing *C*_phos_. Under these conditions, phosphate is predominantly divalent (cf. Figure 3), which can promote dendrimer compaction, analogous to the collapse induced by multivalent ions in linear^68^ and star-like polyelectrolytes.^69,70^ The comparatively weak effect observed here likely reflects both the lower charge of the dendrimer, which reduces ion-correlation effects,^71,72^ and the presence of charge regulation in both the polyelectrolyte and the ions.^64^

To study the effect of phosphate on the local structure of PAMAM, we calculate the local concentration of PAMAM amine monomers

$$c_{\mathrm{mon}}\left(r\right)=C_{\mathrm{mon}}\;g_{\mathrm{mon}}\left(r\right)$$ 18

where *g*_mon_(*r*) is the radial distribution function of the amine monomers to the center of the dendrimer and *C*_mon_ ≈ 39 mM is their bulk concentration. At low pH (red curves), *c*(*r*) exhibits some structure at short distances and extends to larger radii, consistent with stronger intramolecular electrostatic repulsion that drives monomers apart and enhances internal ordering. At intermediate and high pH, the *c*(*r*) profiles become progressively flatter at short distances and show a reduced spatial extension, reflecting the diminished electrostatic repulsion upon the decrease in ionization of PAMAM. No significant dependence on phosphate concentration is observed in the *c*(*r*) profiles, as evidenced by the nearly overlapping curves at each pH.

Overall, these results indicate that while phosphate can significantly modulate the effective charge state of the dendrimer, its impact on the global conformation of PAMAM remains comparatively modest, although still detectable under specific conditions.

**Figure 8:**
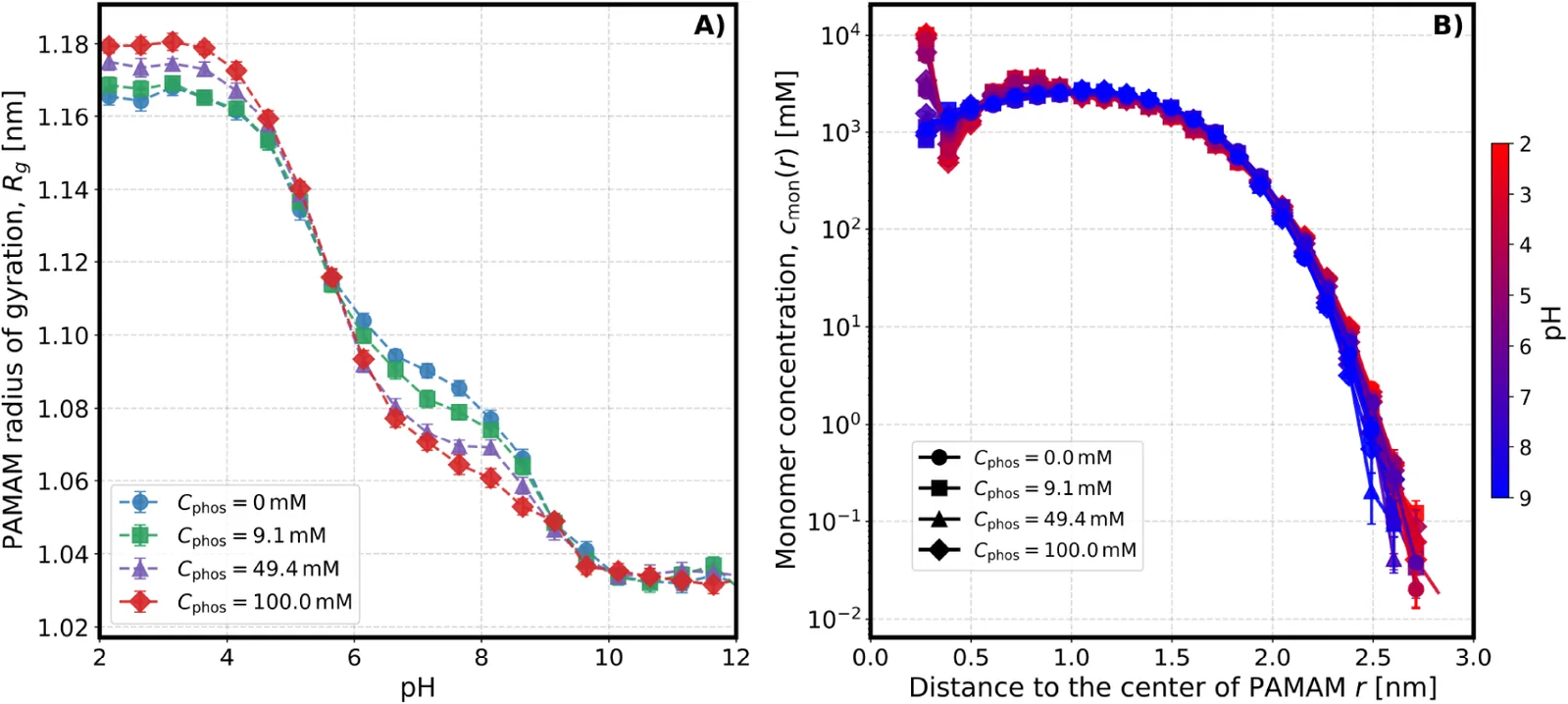
Panel A: Radius of gyration, *R_g_*, of PAMAM as a function of pH for different phosphate concentrations *C*_phos_. Panel B: Local concentration of PAMAM amine monomers *c*(*r*) at distance *r* to the center of the dendrimer for selected pH values (colors) and phosphate concentrations *C*_phos_ (markers).

## 5 Conclusions

In this work, we investigated whether phosphate accumulation around PAMAM dendrimers requires phosphate-specific interactions or can emerge solely from electrostatics and charge regulation. Combining constant-pH simulations, Site Binding theory, and potentiometric titrations, we show that electrostatics and coupled acid−base equilibria alone are sufficient to produce substantial phosphate accumulation and a marked reduction in the effective charge of the dendrimer.

The simulations reveal a strongly asymmetric charge regulation response. While phosphate has only a minor influence on the ionization state of PAMAM, the positively charged dendrimer significantly enhances phosphate ionization in its vicinity. This coupling promotes phosphate accumulation under acidic and near-neutral conditions and leads to substantial charge renormalization of the resulting PAMAM−phosphate complex, even at phosphate concentrations comparable to those used in PBS buffer solutions. At the same time, the structure of the dendrimer is affected only modestly, indicating that the dominant consequences of phosphate association are electrostatic rather than conformational.

The qualitative agreement between our simulations and previous experimental observations of phosphate association with PAMAM dendrimers is particularly noteworthy because the present model intentionally excludes phosphate-specific short-range interactions. This suggests that a substantial fraction of the experimentally observed phosphate accumulation can be understood as a generic consequence of electrostatic attraction and charge regulation, with chemically specific interactions providing additional quantitative contributions rather than being the primary driving force.

More broadly, our results highlight that multivalent buffer ions should not always be regarded as passive pH-control agents or simple screening species. Because their ionization state can itself respond to the local electrostatic environment, buffer ions may actively participate in regulating the charge, interactions, and assembly of weakly ionizable macromolecules. Explicitly accounting for this coupling may therefore be important for understanding and designing biomolecular and soft-matter systems ranging from drug- and gene-delivery vehicles to protein, polyelectrolyte, and colloidal assemblies.

## Supplementary Material



## Notes

Additional details on the coarse-grained molecular simulation model, Langevin Dynamics integration, and constant-pH Monte Carlo sampling. It also describes the experimental potentiometric titration protocols for PAMAM dendrimers and phosphate solutions. Additional sections include a simulation-based comparison between mixed PAMAM−phosphate titration curves and additive reference curves, the calculation of effective phosphate pKa values using a revised implementation of the semiempirical framework by Daniele et al.,^61^ and the fitting of phosphate pKa values from constant-pH simulations. The Supporting Information further provides supplementary figures comparing PAMAM simulations with Site Binding theory, showing the phosphate contribution to the ionic strength of the system, and analyzing the effects of phosphate concentration on charge-regulation capacity and phosphate spatial distributions around the PAMAM dendrimer (PDF)
