# Supplementary material for: Electrostatics and Charge Regulation Drive Phosphate Accumulation in Cationic Dendrimers
Source: ACS Appl Polym Mater. 2026 Jul 31;8(15):13153–66. doi: 10.1021/acsapm.6c02186 (PMC13491548; doi:10.1021/acsapm.6c02186)
Supplement: Supplementary file 1 [file ap6c02186_si_001.pdf]

Supporting Information: Electrostatics and charge regulation drive phosphate accumulation in cationic dendrimers

Pablo M. Blanco,[*,†] Ganesh Balaji,[‡,¶] Corinna Dannert,[‡] Sebastian P. Pineda,[§] Carlos Rey-Castro,[†] Josep Lluís Garcés,[†] Peter Košovan,[§] and Rita S. Dias[*,‡]

†*Department of Chemistry, Physics, Environmental and Soil Sciences, University of Lleida – AGROTECNIO-CERCA Center, Rovira Roure 191, 25198 Lleida, Spain*

‡*Department of Physics, NTNU - Norwegian University of Science and Technology, NO-7491 Trondheim, Norway*

¶*Department of Chemical Engineering, Indian Institute of Technology Madras, IIT P.O., Sardar Patel Road, AdyarChennai, 600036 Tamil Nadu, India*

§*Department of Physical and Macromolecular Chemistry, Faculty of Science, Charles University, Hlavova 2030, 128 40 Prague 2, Czech Republic*

E-mail: pablomiguel.blanco@udl.cat; rita.dias@ntnu.no

S1 Additional details on molecular simulations

S1.1 Potential energy of the system

The coarse-grained model includes bonded, steric, and electrostatic interactions. The total potential energy is expressed as

$$U = U_{\text{bond}} + U_{\text{LJ}} + U_{\text{elec}}. \tag{S1}$$

Bonded PAMAM beads are connected through harmonic springs,

$$U_{\text{bond}} = \frac{1}{2} k \left(r - r_0\right)^2, \tag{S2}$$

where r is the distance between bonded beads, $k = 560\, k_{\text{B}}T/\sigma^2 \approx 6.17 \times 10^4\, \text{kJ}\,\text{mol}^{-1}\,\text{nm}^{-2}$ is the bond force constant, and $r_0 = 0.15\,\text{nm}$ is the equilibrium bond length. This value approximately corresponds to the length of carbon–carbon and carbon–nitrogen single covalent bonds and preserves the spatial separation between neighboring amine sites, which is essential for a realistic description of electrostatic interactions within PAMAM. Since r_0 is the smallest characteristic length scale in the system, it is used as the unit of length in the reduced-unit representation employed throughout the simulations, such that $r_0 = 1$.

Steric non-bonded interactions are modeled using a pairwise augmented Lennard–Jones (LJ) potential,[S1]

$$U_{\text{LJ}}(r) = \begin{cases} \infty, & r < r_{\text{off}}, \\ 4\epsilon \left[\left(\dfrac{\sigma}{r - r_{\text{off}}} \right)^{12} - \left(\dfrac{\sigma}{r - r_{\text{off}}} \right)^{6} \right] - C, & r_{\text{off}} < r < r_{\text{c}}, \\ 0, & r > r_{\text{c}}, \end{cases} \tag{S3}$$

where r is the center-to-center distance between particles i and j, σ defines the interaction

range, ϵ sets the energy scale, r_c is the cutoff distance, and r_{off} is a size-dependent offset. The constant

$$C = 4\epsilon\left[\left(\frac{\sigma}{r_{\text{cut}}}\right)^{12} - \left(\frac{\sigma}{r_{\text{cut}}}\right)^{6}\right] \tag{S4}$$

ensures continuity of the potential at the cutoff, with $r_{\text{cut}} = r_c - r_{\text{off}}$.

We set $\sigma = 1$ for all particle pairs, such that the shape of the LJ interaction is independent of particle size. Differences in particle diameter are instead incorporated through the offset

$$r_{\text{off}} = \frac{1}{2}(d_i + d_j) - \sigma, \tag{S5}$$

where d_i and d_j are the diameters of the interacting particles. This construction ensures that the steric contact distance reflects the physical particle sizes.

For simplicity, PAMAM beads and monovalent ions (Na^+ and Cl^-) are assigned the same diameter, $d_{\text{bead}} = 0.15\,\text{nm} = \sigma$. Phosphate ions are modeled with a larger effective diameter, $d_{\text{phos}} = 0.43\,\text{nm}$, consistent with reported ionic and crystal radii.[S2,S3] The phosphate diameter is assumed to be independent of protonation state, since the reported variations between phosphate species are below the resolution of the present coarse-grained model. We further set $r_c = 2^{1/6}\sigma + r_{\text{off}}$ and $\epsilon = k_BT = 1$, which defines the unit of energy in the reduced-unit system. With this choice of parameters, Eq. S3 reduces to a purely repulsive Weeks–Chandler–Andersen (WCA) potential irrespective of particle size.

Electrostatic interactions between charged particles are described by the Coulomb potential,

$$U_{\text{elec}}(r) = k_BT\, l_B \frac{z_i z_j}{r}, \tag{S6}$$

where z_i and z_j are the particle valencies, k_B is the Boltzmann constant, and l_B is the Bjerrum length,

$$l_B = \frac{e^2}{4\pi\epsilon_0\epsilon_r k_BT}. \tag{S7}$$

The solvent is treated implicitly through the dielectric constant of water, $\epsilon_r = 78.5$, at

$T = 298.15\,\mathrm{K}$. Under these conditions, the Bjerrum length is $l_\mathrm{B} = 0.714\,\mathrm{nm}$. Electrostatic interactions were evaluated under periodic boundary conditions using the particle–particle particle–mesh (P3M) algorithm implemented in ESPResSo.[S4] The algorithm was tuned to the relative accuracy of 10^{-3}, using the automated tuning routine[S5,S6] implemented in the software ESPResSo v4.2.2.

S1.2 Langevin Dynamics

Configurational sampling was performed by integrating the Langevin equation of motion,

$$m\frac{\mathrm{d}\mathbf{v}}{\mathrm{d}t} = -\gamma\mathbf{v} + \sqrt{2\gamma k_\mathrm{B}T}\,\boldsymbol{\xi} - \nabla\mathbf{U}(\mathbf{x}), \tag{S8}$$

where m and $\mathbf{v}$ are the particle mass and velocity, respectively, γ is the damping (Stokes) coefficient, and $\mathbf{U}(\mathbf{x})$ is the total potential energy arising from the molecular interactions described in Sec. S1.1. The thermal energy is given by $k_\mathrm{B}T$, where k_B is the Boltzmann constant and T is the temperature.

The stochastic term $\boldsymbol{\xi}$ represents Gaussian white noise originating from collisions with the implicit solvent and satisfies

$$\langle \boldsymbol{\xi}(t) \rangle = 0, \tag{S9}$$

$$\langle \boldsymbol{\xi}(t) \cdot \boldsymbol{\xi}(t') \rangle = d\,\delta(t - t'), \tag{S10}$$

where $d = 3$ is the dimensionality of the system, δ is the Dirac delta function, and $\langle \cdots \rangle$ denotes an ensemble average. Together, the dissipative and stochastic terms reproduce the Brownian motion of particles in an implicit solvent and ensure canonical sampling at equilibrium.

Simulations were performed in reduced units with thermal energy $k_\mathrm{B}T = \epsilon = 1$, where ϵ defines the unit of energy. The equations of motion were integrated using a Velocity–Verlet

scheme with time step $\Delta t = 0.005\tau$ and damping coefficient $\gamma = 10\,\tau^{-1}$, where $\tau = \sigma\sqrt{m/\epsilon}$ is the unit of time. These parameters were chosen to ensure stable and efficient sampling of configurational space. The particle mass was set to $m = 1$, as it does not affect equilibrium thermodynamic properties.

S1.3 Constant pH Monte Carlo

Titration Monte Carlo moves in the constant-pH ensemble change the protonation state of phosphate and amine groups of the dendrimer. These trial moves are accepted with probability given by the constant-pH ensemble:[S7]

$$P_{\mathrm{cpH}} = \min\left\{1,\, \exp\left[-\frac{\Delta U}{k_B T} + \xi\ln(10)\left(\mathrm{pH}^{\mathrm{input}} - \mathrm{p}K_{\mathrm{A}}\right)\right]\right\}, \tag{S11}$$

where $\mathrm{p}K_{\mathrm{A}} = -\log K_a$, and ΔU is the change in potential energy upon the trial move, typically dominated by electrostatic contributions. The parameter ξ describes the direction of the reaction: $\xi = 1$ for protonation and $\xi = -1$ for deprotonation.

Following the Addition of a Cation Procedure (ACP),[S8] protonation and deprotonation moves are coupled to the creation or deletion of a cation in order to preserve electroneutrality. The activity coefficient of the cation, $\gamma_{\mathrm{Na^+}}$, is accounted for a posteriori by shifting the pH scale:[S9]

$$\mathrm{pH} = \mathrm{pH}^{\mathrm{input}} + p\gamma_{\mathrm{Na^+}}, \tag{S12}$$

where $p\gamma_{\mathrm{Na^+}} = -\log\gamma_{\mathrm{Na^+}}$. The activity coefficient is estimated using the semi-empirical Davies equation:

$$p\gamma_{\mathrm{Na^+}} = A\left(\frac{\sqrt{I/c^{\ominus}}}{1+\sqrt{I/c^{\ominus}}} - C\frac{I}{c^{\ominus}}\right), \tag{S13}$$

where $A \approx 0.509$ and $C = 0.3$ are empirical parameters for water at 298 K, I is the ionic strength, and $c^{\ominus} = 1\ \mathrm{mol\,kg^{-1}}$ is the standard concentration.

S2 Additional details on the protocol for potentiometric titration

S2.1 Titration protocol and sample preparation

S2.1.1 PAMAM

PAMAM dendrimers generation 2.0 (G2) with ethylenediamine cores (supplied as 20 wt% in methanol, purchased from Sigma Aldrich/Merck) were, prior to the titration, dialyzed against pure MilliQ water overnight at room temperature, and freeze-dried. We performed the potentiometric titrations on a Metrohm 888 Titrando Compact titrator equipped with a Pt1000 temperature sensor and a magnetic stirrer. The pH was measured using a LL Biotrode 3.0 mm glass electrode and the results were collected by Titrando Software. Sample preparation consisted of weighing 18.0 mg of dendrimer and dissolving them independently in $V_0 = 1.7\,\mathrm{mL}$ of standardized HCl (0.1 M). Potentiometric titrations were performed at 23 °C from a pH ≈ 1 by gradual addition of standardized NaOH (0.1 M) until reaching a pH ≈ 12.5. The pH meter signal drift was limited to $0.1\,\mathrm{mV\,s^{-1}}$, with a maximum interval of 100 s between titrant additions to allow equilibration. The blank titration consisted of titrating 2.0 mL of standardized HCl (0.1 M) using a standardized NaOH (0.1 M) under the same conditions as the dendrimer titrations. For this titration, the blank correction was not applied due to an insufficient number of data points in the blank titration curve to enable a reliable subtraction. However, visual inspection of the blank curve confirmed that its contribution is small and varies smoothly across the titration window. Standardized HCl and NaOH solutions were prepared from ampules (Penta s.r.o.) designed for 1000 mL 0.1 M volumetric solutions and stored under soda lime to prevent CO_2 absorption. All solutions were prepared with Milli-Q water produced by a Barnstead MicroPure water purification system (Thermo Scientific).

S2.1.2 Phosphate solution

We performed the potentiometric titrations on a ProbeDrum titrator with temperature control and equipped with a magnetic stirrer. The pH was measured using an advanced electrochemistry meter Orion Versastar (Thermo Scientific) using a Orion Micro Glass Bodied Combination pH Electrode (8220BNWP), and the results were collected by ProbEx Software. Sample preparation consisted of weighing 3.4 mg of Na_3PO_4 (96% purity, Sigma-Aldrich) and dissolving it in 2.0 mL of standardized NaOH (0.1 M) under an argon atmosphere to prevent CO_2 absorption. This yielded a total phosphate concentration of approximately (10 mM) and an initial pH value $pH_0 = 13.028$. Prior the actual titration, 229.085 µL of standardized HCl (1 M) was added to lower the pH to 10.964, giving $V_0 = 2229.085$ µL. The potentiometric titration was then performed at 20 °C by the addition of 0.1 µL step volumes of standardized HCl (1 M) until reaching a pH = 2.846. The maximum interval between titrant additions was 40 s to allow equilibration, and the total volume of titrant added during the titration was 34.812 µL. The blank titration consisted of titrating 2.0 mL of standardized NaOH (0.1 M) with standardized HCl (1 M), following the same protocol as the phosphate titrations. For this titration, the blank correction was applied through interpolation of the blank titration curve, which contained sufficient data points to reliably match the pH values of the phosphate titration. Standardized HCl and NaOH solutions were prepared from ampules (Sigma-Aldrich) designed for 1000 mL 1 M and 0.1 M volumetric solutions, respectively. The NaOH solution was prepared using degassed water and stored under an argon atmosphere to prevent CO_2 absorption. All solutions were prepared with water produced by a Milli-Q Advantage A10 water purification system (Thermo Scientific).

The resulting titration curves are shown in Fig. S1, where the pH is plotted as a function of the added HCl volume for both the blank and phosphate titrations.

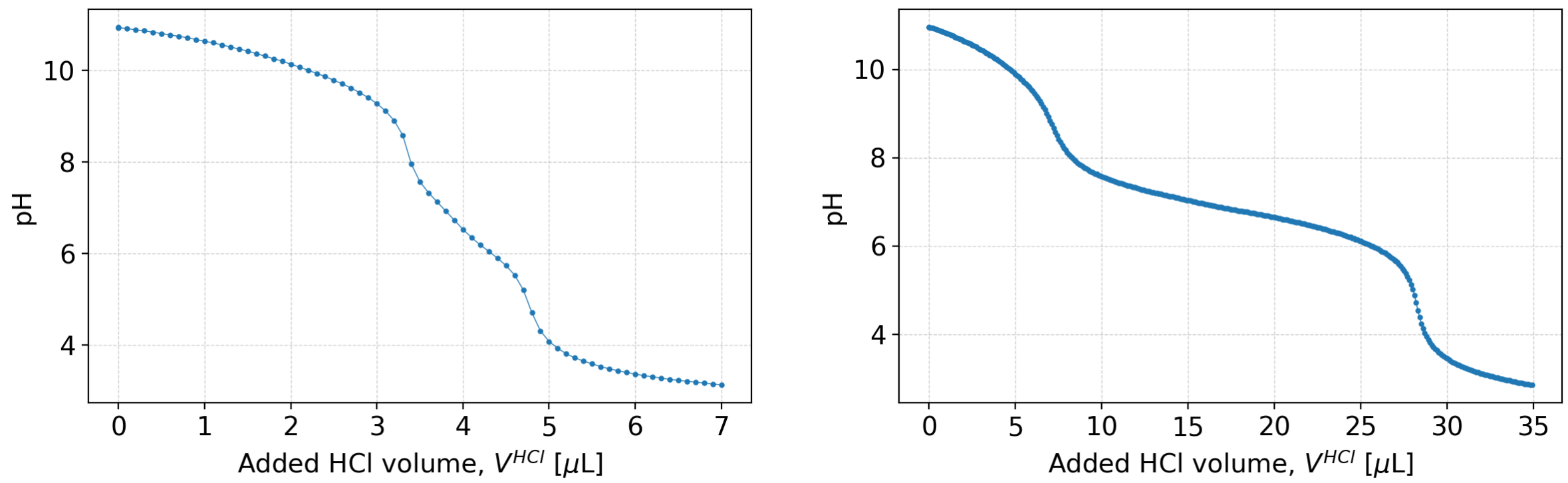

Figure S1: Titration curves for the blank (left) and 10 mM phosphate (right) solutions, showing pH as a function of added HCl volume.

S2.2 Data analysis

The raw data collected by the potentiometric titrations correspond to a set of pH values, added titrant volume (V^{NaOH} for the PAMAM titration and V^{HCl} for the phosphate titration) and temperatures at different time intervals. Using this information, we calculated the net charge as follows,

$$Q^0_{\mathrm{analyte}} = (-1) \cdot \frac{(n_Q^{\mathrm{NaOH}} - n_Q^{\mathrm{HCl}} + n_Q^{\mathrm{OH}^-} - n_Q^{\mathrm{H}^+})}{n_{\mathrm{analyte}}} + Q_{\mathrm{extra}}, \tag{S14}$$

where n_{analyte} is the number of moles of analyte and Q_{extra} is a shift due to unaccounted charges in solution. Here, n_Q^{NaOH} and n_Q^{HCl} are molar equivalents contributed by NaOH and HCl, respectively, calculated as:

$$n_Q^{\mathrm{NaOH}} = V^{\mathrm{NaOH}} \cdot c_{\mathrm{NaOH}}, \qquad n_Q^{\mathrm{HCl}} = V^{\mathrm{HCl}} \cdot c_{\mathrm{HCl}}. \tag{S15}$$

We assumed negligible the analyte volume, then $V^{\mathrm{HCl}} = V_0$ for PAMAM titrations and $V^{\mathrm{NaOH}} = V_0$ for phosphate titrations. The molar equivalents of H^+ and OH^- ions at each step of the titration were calculated from the pH and the pK_{w} values at the registered temperatures, as follows:

$$n_Q^{\mathrm{H}^+} = \frac{10^{(-\mathrm{pH})} c^{\ominus} V_{\mathrm{total}}}{\gamma_\pm}, \qquad n_Q^{\mathrm{OH}^-} = \frac{10^{(\mathrm{pH}-\mathrm{p}K_{\mathrm{w}})} c^{\ominus} V_{\mathrm{total}}}{\gamma_\pm}, \tag{S16}$$

here, V_{total} is the total volume at each titration step: $V_{\mathrm{total}} = V_0 + V^{\mathrm{NaOH}}$ for PAMAM titrations and $V_{\mathrm{total}} = V_0 + V^{\mathrm{HCl}}$ for phosphate titrations. The reference concentration is $c^{\ominus} =$ 1.0 mol/kg which can be approximated as 1.0 M in dilute aqueous solutions. Additionally, we assumed the activity coefficient of small ions ($\gamma_\pm$) equal to unity, which introduces a negligible error in the net charge calculation since the $n_Q^{\mathrm{H}^+}$ and $n_Q^{\mathrm{OH}^-}$ correction terms are only significant at extreme pH values (pH $<$ 2.0 or pH $>$ 12.0), outside the titration window. We took the temperature-dependence of pK_{w} into account, following Ref.[S10]

The values of Q_{extra} and n_{analyte} are generally difficult to determine precisely due to unknown sample purity or excipient composition. To place the experimentally reconstructed charge on an absolute scale, we applied a linear calibration based on two reference points corresponding to well-defined protonation states. This procedure maps the raw charge obtained from Eq. S14 onto the expected theoretical charge scale.

We define an affine transformation of the form

$$Q_{\mathrm{analyte}} = s Q^0_{\mathrm{analyte}} + Q_{\mathrm{o}}, \tag{S17}$$

where Q^0_{analyte} is the uncalibrated net charge per mole of analyte, and s and Q_{o} are a scaling factor and an offset, respectively.

The parameters are determined by enforcing consistency with two reference plateau regions, denoted P1 and P2, corresponding to low- and high-pH limits:

$$s = \frac{Q_{\mathrm{P1}} - Q_{\mathrm{P2}}}{Q^0_{\mathrm{analyte}}(\mathrm{P1}) - Q^0_{\mathrm{analyte}}(\mathrm{P2})}, \tag{S18}$$

$$Q_{\mathrm{o}} = Q_{\mathrm{P1}} - s Q^0_{\mathrm{analyte}}(\mathrm{P1}). \tag{S19}$$

Here, $Q^0_{\mathrm{analyte}}(\mathrm{P1})$ and $Q^0_{\mathrm{analyte}}(\mathrm{P2})$ are the experimentally estimated charges evaluated

at the pH values defining the two plateau regions, while Q_{P1} and Q_{P2} are the corresponding theoretical charges at well-defined reference charge states estimated from the expected acid–base speciation at the corresponding pH values. The scale factor s corrects the amplitude of the charge variation between the two reference plateaus, and therefore accounts collectively for uncertainties in the nominal analyte amount, titrant concentration, sample hydration or salt form, residual blank contributions, and the choice of reference plateau regions. Similarly, Q_{o} corrects the charge origin and absorbs contributions from initial acid or base, residual counterions, and imperfect blank correction. All calibration parameters are reported in Tab. S1.

Table S1: Affine calibration parameters used to map the experimentally estimated charge Q^0_{analyte} onto the theoretical charge scale.

System	s	Q_{o}	P1 (low pH plateau)	P2 (high pH plateau)
PAMAM	0.9586	116.3419	pH = 3.5625<br>$Q^0_{\mathrm{analyte}} = -90.3941$<br>$Q_{\mathrm{P1}} = 29.6931$	pH = 9.9580<br>$Q^0_{\mathrm{analyte}} = -118.7563$<br>$Q_{\mathrm{P2}} = 2.5061$
Phosphate	1.0049	-2.1815	pH = 4.54<br>$Q^0_{\mathrm{analyte}} = 1.1757$<br>$Q_{\mathrm{P1}} = -1.0$	pH = 9.01<br>$Q^0_{\mathrm{analyte}} = 0.1806$<br>$Q_{\mathrm{P2}} = -2.0$

S3 Simulation-based comparison of mixed and additive titration curves

To assess whether a potentiometric titration of a mixed PAMAM–phosphate solution would provide a sensitive experimental signature of their mutual interaction, we compared two simulated total-charge curves. The first corresponds to the total charge of the mixed PAMAM–phosphate system, calculated directly from simulations in which PAMAM and phosphate coexist in solution,

$$Q_{\mathrm{tot}}^{\mathrm{mix}} = Q_{\mathrm{PAMAM}}^{\mathrm{mix}} + N_{\mathrm{phos}} Q_{\mathrm{phos}}^{\mathrm{mix}}, \tag{S20}$$

where $Q_{\mathrm{PAMAM}}^{\mathrm{mix}}$ is the average PAMAM charge in the mixed system, $Q_{\mathrm{phos}}^{\mathrm{mix}}$ is the average charge per phosphate ion in the mixed system, and N_{phos} is the total number of phosphate ions. The second curve is an additive reference constructed from independent simulations of PAMAM and phosphate,

$$Q_{\mathrm{tot}}^{\mathrm{sep}}(\mathrm{pH}) = Q_{\mathrm{PAMAM}}^{\mathrm{pure}}(\mathrm{pH}) + N_{\mathrm{phos}} Q_{\mathrm{phos}}^{\mathrm{pure}}(\mathrm{pH}). \tag{S21}$$

The residuals, calculated as the difference between both curves,

$$\mathrm{Residuals} = Q_{\mathrm{tot}}^{\mathrm{mix}}(\mathrm{pH}) - Q_{\mathrm{tot}}^{\mathrm{sep}}(\mathrm{pH}), \tag{S22}$$

quantifies the extent to which the mixed titration deviates from the linear superposition of the isolated PAMAM and phosphate titrations.

As shown in Fig. S2, the total charge of the mixed system is very close to the additive reference over the full pH range, with deviations below approximately $0.5e$. This indicates that, although the simulations predict local phosphate accumulation and local changes in phosphate ionization near PAMAM, these effects are largely masked in the total charge of the solution. Consequently, a conventional potentiometric titration of the mixed system is

not expected to provide a sensitive validation of phosphate association or effective-charge reduction.

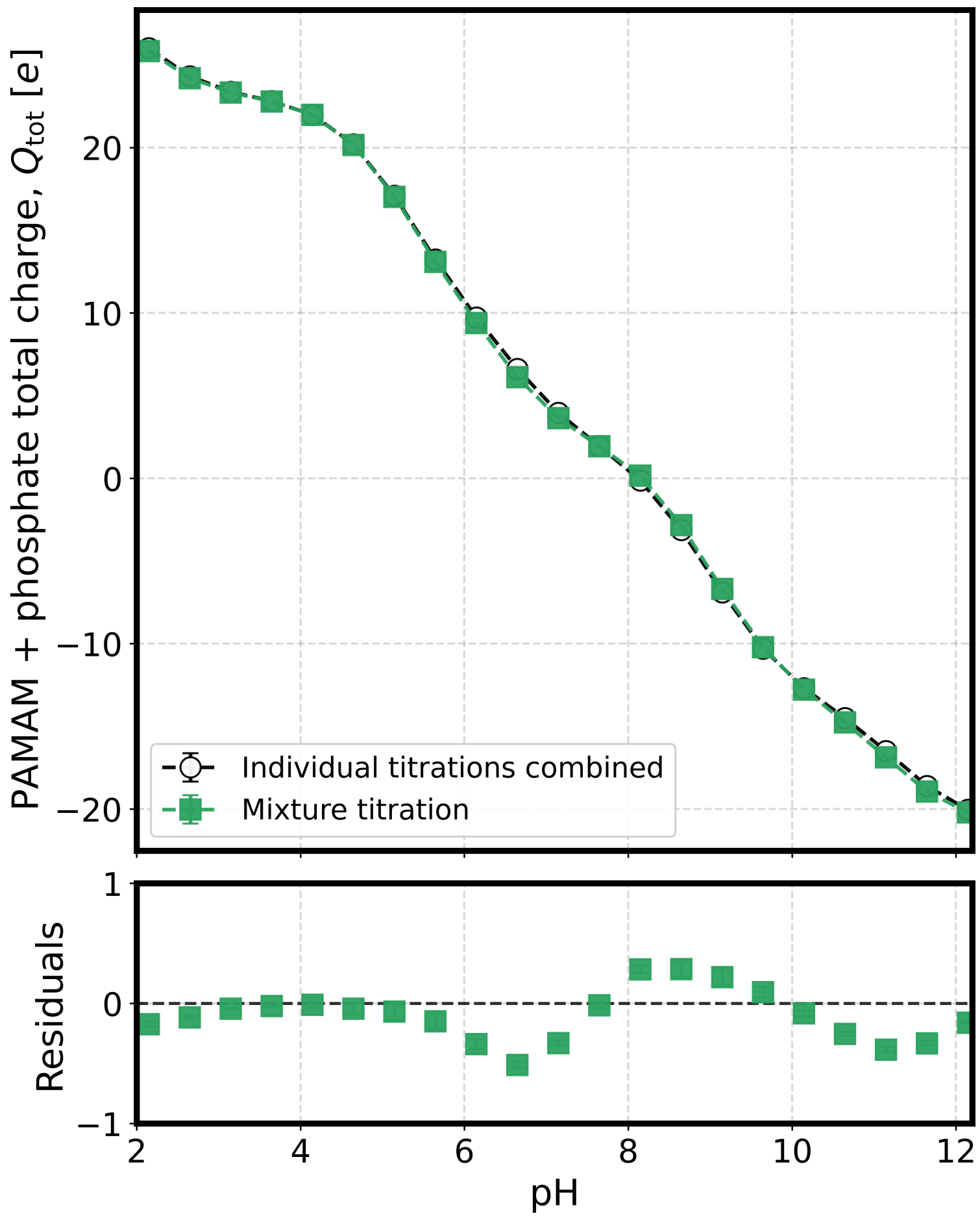

Figure S2: Simulation-based comparison between the total titration charge of the mixed PAMAM–phosphate system and an additive reference constructed from independent PAMAM and phosphate simulations at $C_{\mathrm{phos}} = 9.1\,\mathrm{mM}$. The top panel shows $Q_{\mathrm{tot}}^{\mathrm{mix}}$ (Eq. S20, green squares) together with $Q_{\mathrm{tot}}^{\mathrm{sep}}$ (Eq. S21, empty circles), The bottom panel shows the residuals between both data series (Eq. S22).

S4 Calculation of the effective $\mathrm{p}K_\mathrm{A}$ values of phosphate

Daniele *et al.*[S11] provide a semi-empirical framework to calculate the effective acidity constants of phosphate at various temperatures and in the presence of various background electrolytes. We used the framework proposed by Daniele and co-workers to estimate the effective phosphate acidity constants expected under our experimental and simulation conditions of 0.1 M NaCl at $T = 25\,^\circ\mathrm{C}$.

Daniele *et al.* proposed the following expression to calculate the effective acidity constants

$$\begin{aligned} \log K_j^{\mathrm{eff}}(I,T) = \; & \log K_j^{\mathrm{eff}}(I',\theta) + a_1(T-\theta) + a_2(T-\theta)^2 \\ & - 2(4-j)\left[G(I,T) - G(I',\theta)\right] \\ & + I\left[C_\theta + c_1(T-\theta)\right] + I^{3/2}\left[D_\theta + d_1(T-\theta)\right], \end{aligned} \tag{S23}$$

where

$$G(I,T) = \frac{0.5I}{1+\sqrt{I}\left[B + b_1(T-\theta)\right]}. \tag{S24}$$

Here, I' and θ are the reference ionic strength and temperature. For phosphate, the effective acidity constants are defined as

$$K_j^{\mathrm{eff}} = \frac{[\mathrm{H}_j\mathrm{PO}_4^{j-3}]}{[\mathrm{H}^+][\mathrm{H}_{j-1}\mathrm{PO}_4^{j-4}]}, \tag{S25}$$

The values of all parameters for phosphate in aqueous solution with NaCl as background electrolyte, including the semi-empirical parameters a_1, a_2, B, b_1, C_θ, c_1, D_θ and d_1, are reported in Tab. S2. Daniele *et al.* accounted for the self-association of NaCl by replacing the formal ionic strength I with an effective ionic strength $I_\mathrm{e} = \alpha C_\mathrm{NaCl}$, where C_NaCl is the

concentration of NaCl and the degree of dissociation α is given by

$$\begin{aligned}\alpha = 1 &- C_{\mathrm{NaCl}}^{1/2}\left[0.033 - 9\times10^{-4}(T-25)\right]\\ &- C_{\mathrm{NaCl}}\left[0.219 - 4\times10^{-4}(T-25)\right]\\ &+ 0.079\, C_{\mathrm{NaCl}}^{3/2},\end{aligned} \tag{S26}$$

where C_{NaCl} is expressed in molar units and T in degrees Celsius. For $C_{\mathrm{NaCl}} = 0.1\,\mathrm{M}$ and $T = 25\,°\mathrm{C}$, Eq. S26 gives $I_{\mathrm{e}} = 0.0970\,\mathrm{M}$.

Here, we used a slightly revised form of the semi-empirical framework proposed by Daniele *et al.*[S11] In applying the original equations, we noticed that Eq. S23 does not recover the tabulated reference constants at the reference state, *i.e.* when $I = I'$ and $T = \theta$, because the terms proportional to I and $I^{3/2}$ do not vanish at the reference state. We therefore used the following revised form, in which these terms are expressed relative to the reference ionic strength:

$$\begin{aligned}\log K_j^{\mathrm{eff}}(I,T) = &\ \log K_j^{\mathrm{eff}}(I',\theta) + a_1(T-\theta) + a_2(T-\theta)^2\\ &- 2(4-j)\left[G_{\mathrm{corr}}(I,T) - G_{\mathrm{corr}}(I',\theta)\right]\\ &+ (I - I')\left[C_\theta + c_1(T-\theta)\right]\\ &+ \left(I^{3/2} - I'^{3/2}\right)\left[D_\theta + d_1(T-\theta)\right].\end{aligned} \tag{S27}$$

In addition, we used $\sqrt{I}$ rather than I in the numerator of the Debye–Huckel term, consistent with the form of the Debye–Huckel expression on which the semi-empirical formulation of Daniele *et al.* is based:

$$G_{\mathrm{corr}}(I,T) = \frac{0.5\sqrt{I}}{1 + \sqrt{I}\left[B + b_1(T-\theta)\right]}. \tag{S28}$$

These revisions, as well as the corrected value of the coefficient $10^3 c_1$ for $j = 2$ in Tab. S2, were later confirmed by Prof. Demetrio M. Milea and Prof. Concetta De Stefano in a per-

sonal communication. Using these revised equations (Eqs. S27 and S28), we recovered the tabulated values presented in the original article by Daniele *et al.*,[S11] including the ones in the reference state. Using these revised equations at $I = I_e = 0.0970\,\mathrm{M}$ and $T = 25\,°\mathrm{C}$, we obtain

$$\mathrm{p}K_\mathrm{A}^{\mathrm{eff},1} = 1.914, \qquad \mathrm{p}K_\mathrm{A}^{\mathrm{eff},2} = 6.736, \qquad \mathrm{p}K_\mathrm{A}^{\mathrm{eff},3} = 11.625. \tag{S29}$$

These are the $\mathrm{p}K_\mathrm{A}^{\mathrm{eff}}$ values used to calculate effective HH curve in Fig. 2B in the main text.

Table S2: Values of the semi-empirical coefficients for phosphate in aqueous solution with NaCl as background electrolyte at the reference state $I' = 0.16\,\mathrm{M}$ and $\theta = 25\,°\mathrm{C}$. Values taken from Daniele *et al.*,[S11] , with the corrected value of $10^3 c_1$ for $j = 2$ confirmed by Prof. Demetrio M. Milea and Dr. Concetta De Stefano in a personal communication.

j	$\log K_j^{\mathrm{H}}(I', \theta)$	$10^3 a_1$	$10^5 a_2$	B	$10^3 b_1$	C_θ	$10^3 c_1$	D_θ	$10^3 d_1$
1	11.491	-18.5	26.4	0.946	-2.92	0.231	-5.19	0	0
2	6.649	-2.76	7.03	1.000	0	0.212	-1.3	-0.124	2.16
3	1.870	3.48	3.83	1.000	0	0.066	-4.74	0	0

S5 Comparison of the Site Binding theory with the molecular simulation for the individual titration of G2 PAMAM

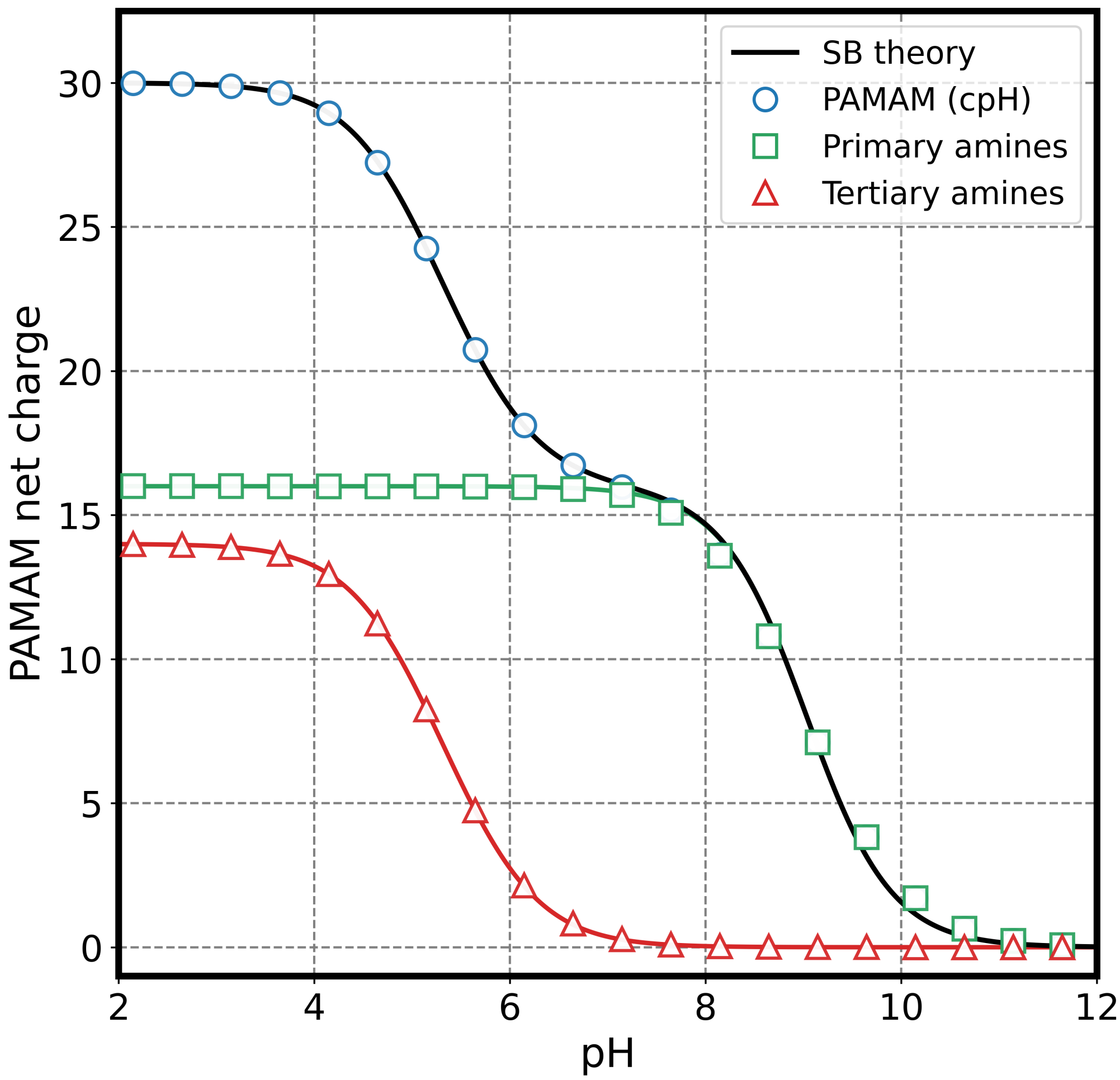

Figure S3: Net charge of PAMAM as a function of pH obtained from cpH simulations (symbols). The black line shows the theoretical curve fitted to the simulation data, yielding $\mathrm{p}K_{\mathrm{I}} = 9.03$, $\mathrm{p}K_{\mathrm{III}} = 5.54$, and $\varepsilon = 0.104$. The green and red lines denote the primary and tertiary contributions to the net charge predicted from SB theory using the same parameters without any further fitting.

S6 Fitting of the effective pKa values of phosphate from cpH simulation

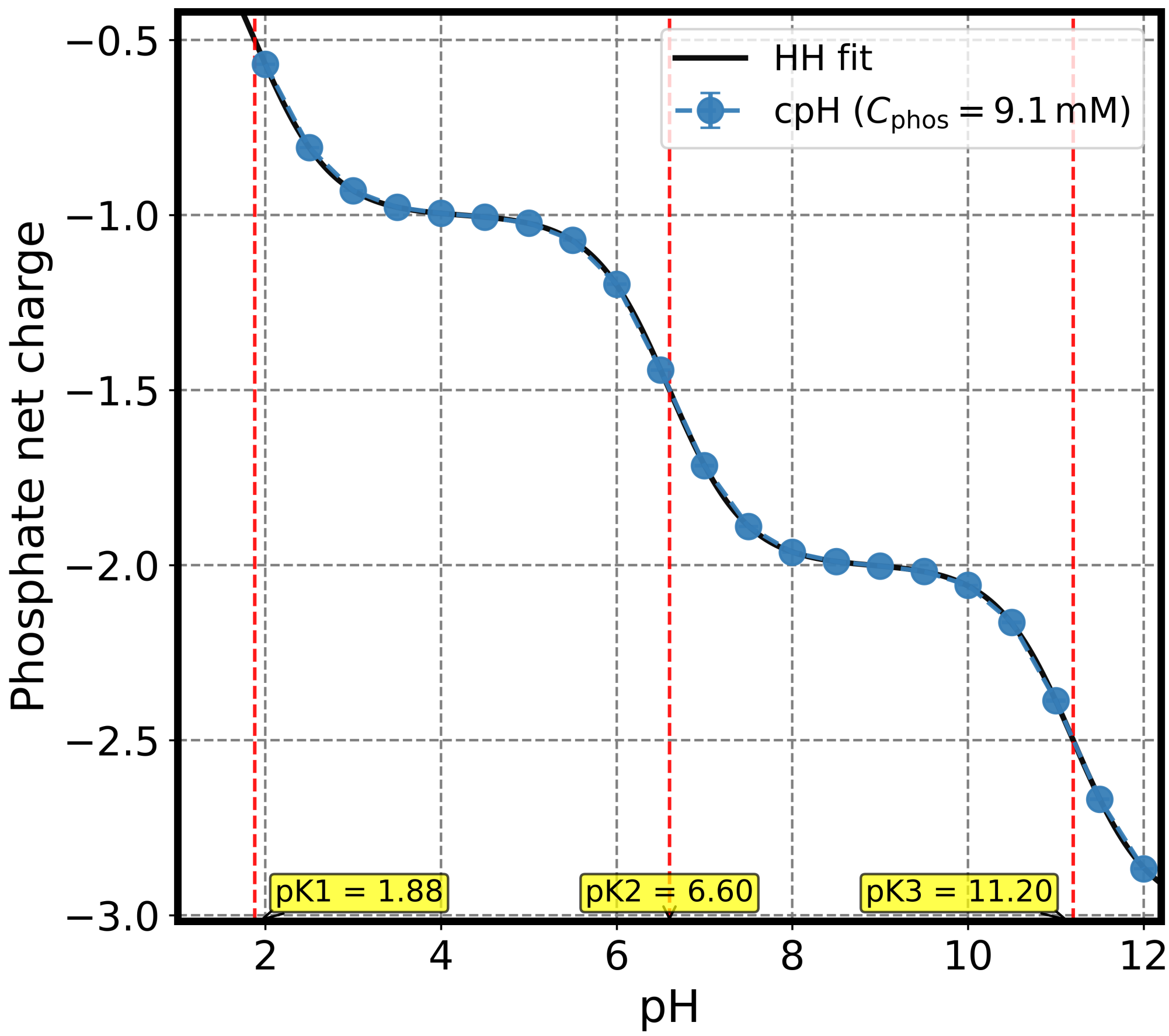

Figure S4: Net charge of phosphate as a function of pH obtained from cpH simulations (symbols). The solid line shows the Henderson–Hasselbalch fit used to determine the effective acidity constants, pK_a, reported in the figure.

S7 Effect of phosphate on the ionic strength of the system

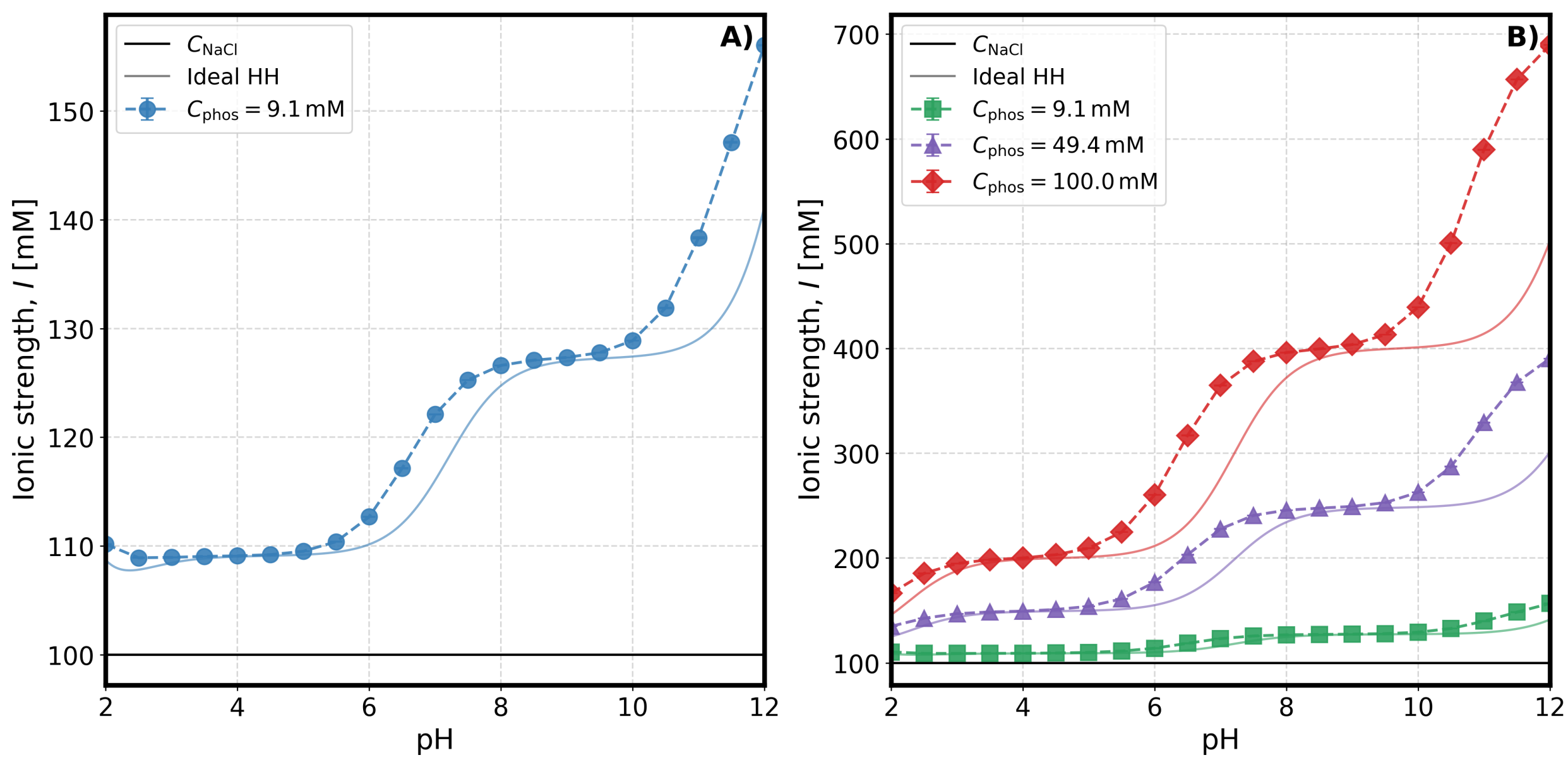

Figure S5: Ionic strength I of the phosphate only solution (Panel A) and PAMAM-containing phosphate solutions (Panel B) as a function of pH for different total phosphate concentrations, C_{phos}. I was calculated from the small-ion composition as $I = \frac{1}{2}\sum_i c_i z_i^2$. Symbols correspond to constant-pH simulation data, while solid colored lines show ideal Henderson–Hasselbalch predictions for phosphate speciation. The black horizontal line marks the nominal NaCl background concentration, $C_{\mathrm{NaCl}} = 100\,\mathrm{mM}$. Even at the lowest phosphate concentration studied, $C_{\mathrm{phos}} = 9.1\,\mathrm{mM}$, phosphate increases the total ionic strength above the background salt level, with a stronger contribution at high pH due to phosphate deprotonation.

S8 Effect of phosphate concentration in the charge regulation capacity

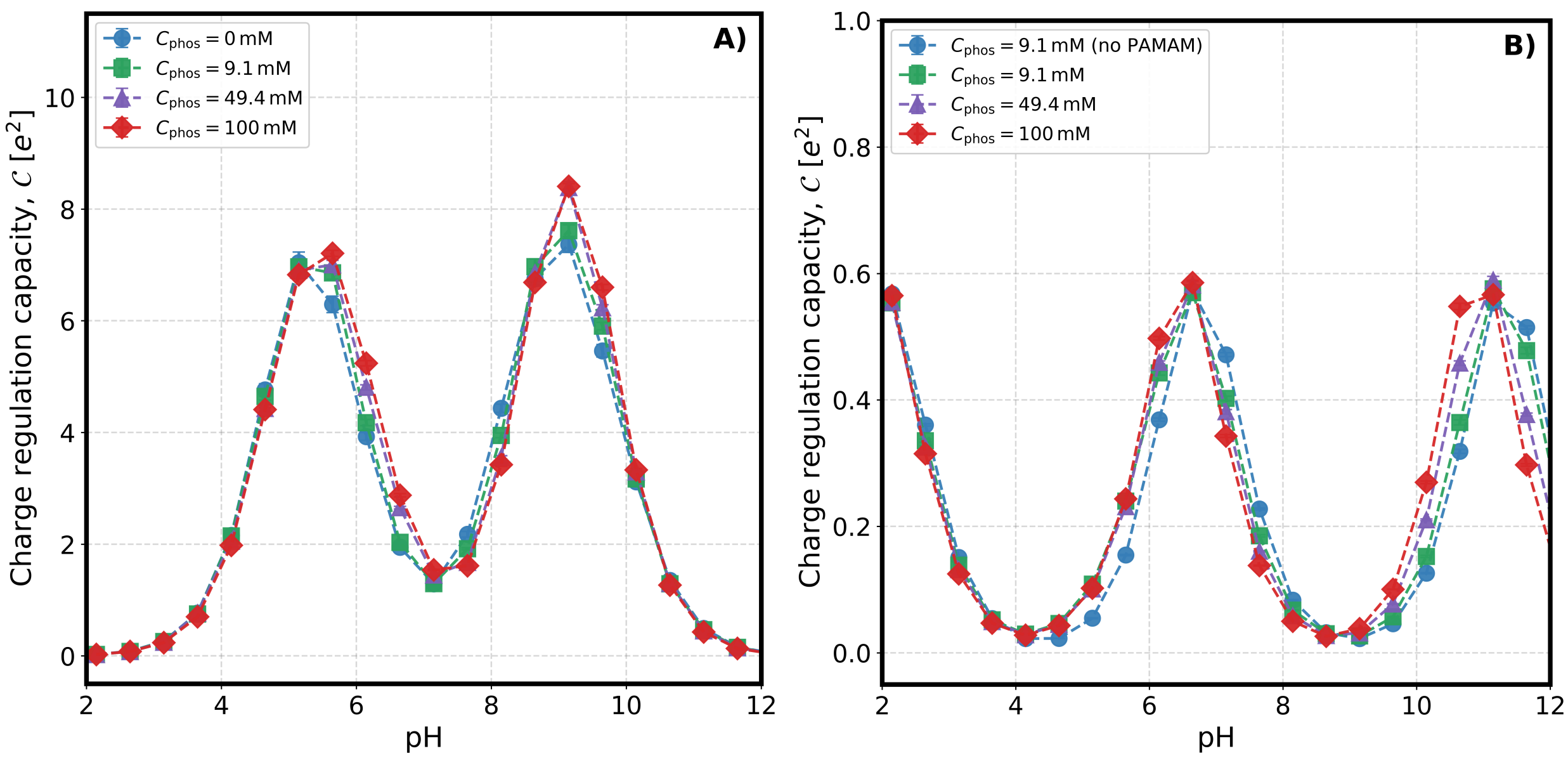

Figure S6: Charge regulation capacity $\mathcal{C}$ of PAMAM (panel A) and phosphate (panel B) together in solution at various phosphate concentrations C_{phos} obtained by cpH simulation. For reference, data from phosphate in bulk solution (in absence of PAMAM) at $C_{phos} = 9.1$ mM is included (blue circles).

S9 Additional phosphate distributions around PAMAM at various phosphate concentration

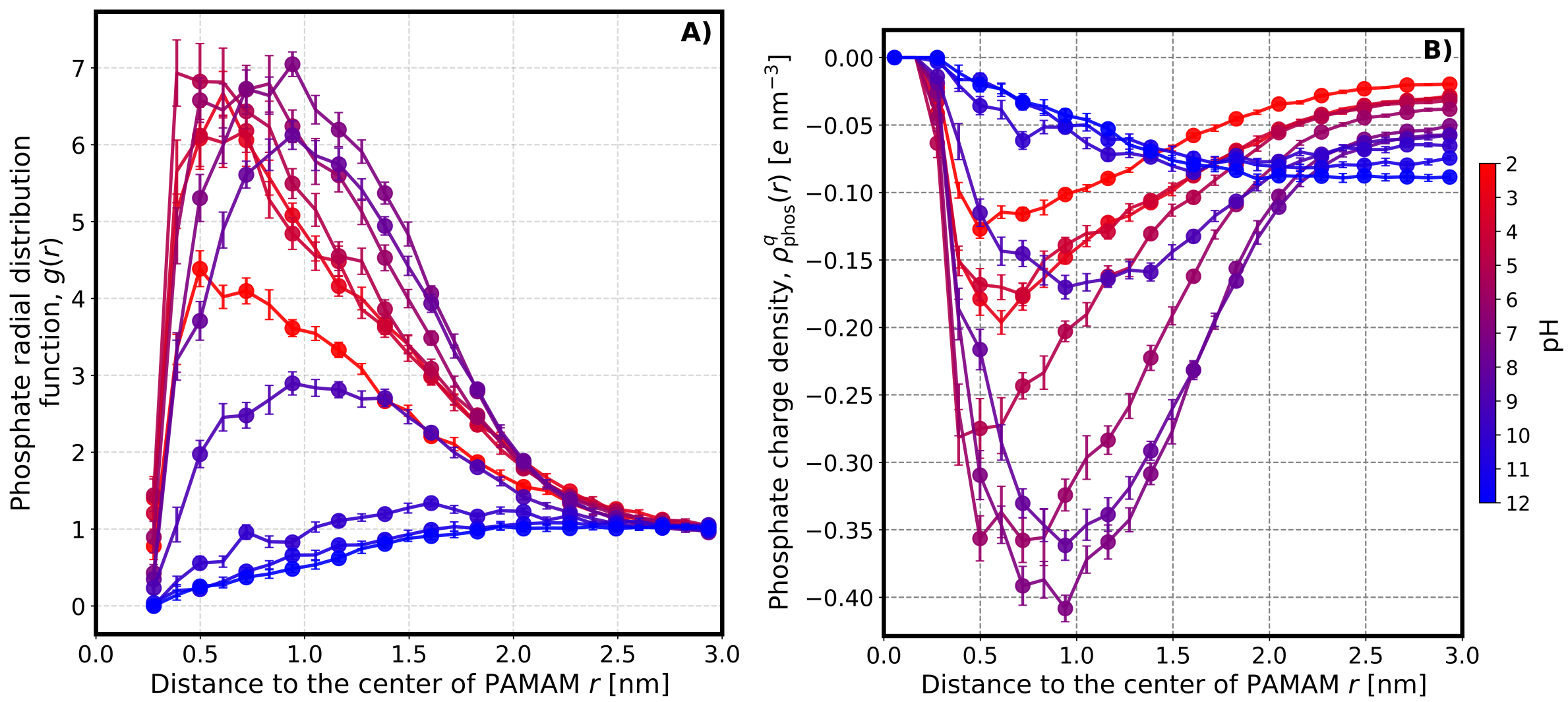

Figure S7: Phosphate spatial distributions around PAMAM at $C_{\mathrm{phos}} = 49\,\mathrm{mM}$ for selected pH values. Panel A: radial distribution function $g(r)$ of phosphate ions around the center of the dendrimer. Panel B: phosphate charge density $\rho(r)$ as a function of the distance to the dendrimer center. The color gradient ranges from pH = 2 (red) to pH = 12 (blue).

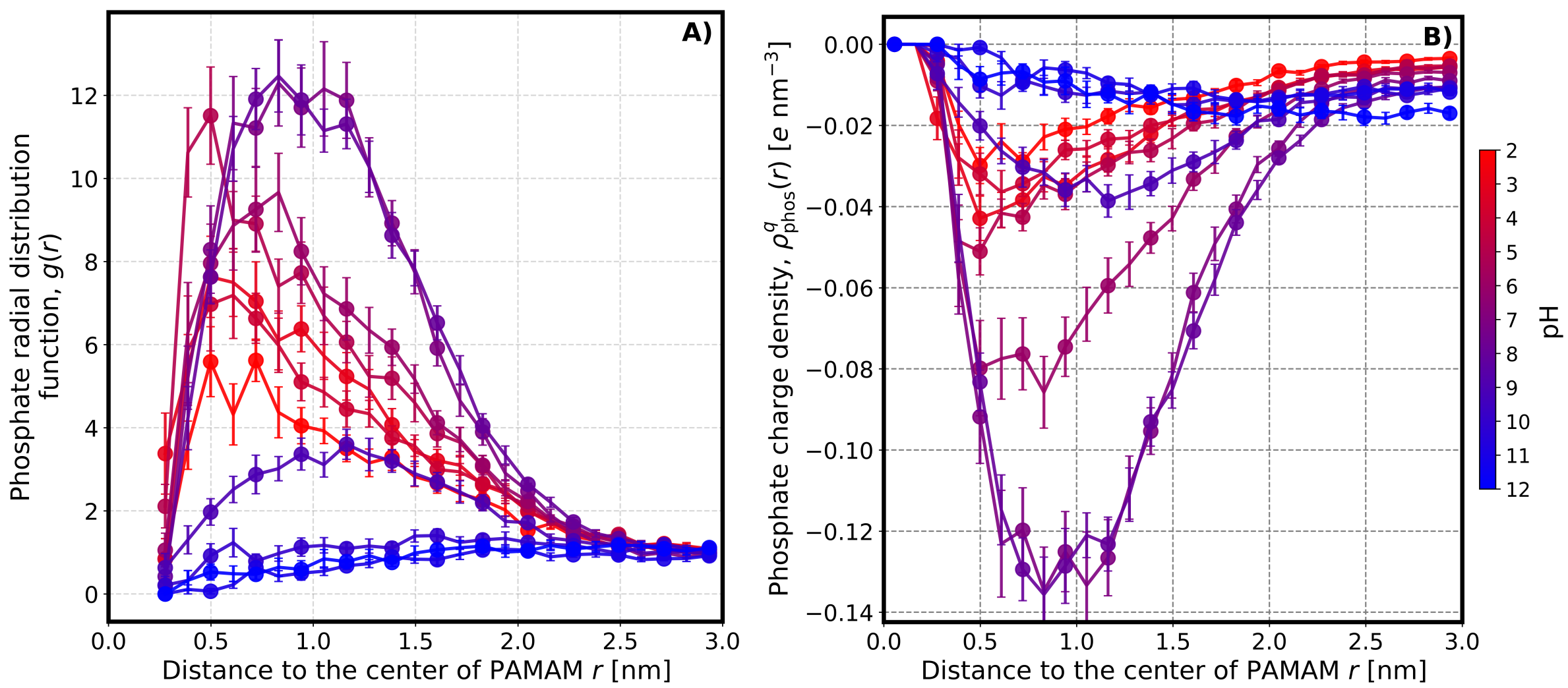

Figure S8: Phosphate spatial distributions around PAMAM at $C_{\text{phos}} = 9.1\,\text{mM}$ for selected pH values. Panel A: radial distribution function $g(r)$ of phosphate ions around the center of the dendrimer. Panel B: phosphate charge density $\rho(r)$ as a function of the distance to the dendrimer center. The color gradient ranges from pH = 2 (red) to pH = 12 (blue).

References

(S1) Beyer, D.; Torres, P. B.; Pineda, S. P.; Narambuena, C. F.; Grad, J.-N.; Košovan, P.; Blanco, P. M. pyMBE: The Python-based molecule builder for ESPResSo. *The Journal of Chemical Physics* **2024**, *161*, 022502.

(S2) Marcus, Y. Ionic Radii in Aqueous Solutions. *Chemical Reviews* **1988**, *88*, 1475–1498.

(S3) Kiriukhin, M. Y.; Collins, K. D. Dynamic Hydration Numbers for Biologically Important Ions. *Biophysical Chemistry* **2002**, *99*, 155–168.

(S4) Hockney, R. W.; Eastwood, J. W. *Computer simulation using particles*; Taylor & Francis, New York, 1988.

(S5) Deserno, M.; Holm, C. How to mesh up Ewald sums. I. A theoretical and numerical comparison of various particle mesh routines. *The Journal of chemical physics* **1998**, *109*, 7678–7693.

(S6) Deserno, M.; Holm, C. How to mesh up Ewald sums. II. An accurate error estimate for

the particle–particle–particle-mesh algorithm. *The Journal of chemical physics* **1998**, *109*, 7694–7701.

(S7) Reed, C. E.; Reed, W. F. Monte Carlo study of titration of linear polyelectrolytes. *The Journal of Chemical Physics* **1992**, *96*, 1609–1620.

(S8) Labbez, C.; Jönsson, B. In *Applied Parallel Computing. State of the Art in Scientific Computing*; Kågström, B., Elmroth, E., Dongarra, J., Waśniewski, J., Eds.; Lecture Notes in Computer Science; Springer Berlin Heidelberg, 2007; Vol. 4699; pp 66–72.

(S9) Beyer, D.; Blanco, P. M.; Landsgesell, J.; Košovan, P.; Holm, C. How To Correct Erroneous Symmetry-Breaking in Coarse-Grained Constant-pH Simulations. *Journal of Chemical Theory and Computation* **2025**, *21*, 1396–1404.

(S10) Bandura, A. V.; Lvov, S. N. The Ionization Constant of Water over Wide Ranges of Temperature and Density. *Journal of Physical and Chemical Reference Data* **2006**, *35*, 15–30.

(S11) Daniele, P. G.; De Robertis, A.; De Stefano, C.; Gianguzza, A.; Sammartano, S. Salt effects on the protonation of ortho-phosphate between 10 and 50 C in aqueous solution. A complex formation model. *Journal of solution chemistry* **1991**, *20*, 495–515.
